# Variability and uncertainty in forest biomass estimates from the tree to landscape scale: the role of allometric equations

**DOI:** 10.1186/s13021-020-00143-6

**Published:** 2020-05-14

**Authors:** Anthony G. Vorster, Paul H. Evangelista, Atticus E. L. Stovall, Seth Ex

**Affiliations:** 1 Natural Resource Ecology Laboratory, Colorado State University, Fort Collins, CO 80523 USA; 2 Graduate Degree Program in Ecology, Colorado State University, Fort Collins, CO 80523 USA; 3 NASA Goddard Space Flight Center, Greenbelt, MD 20771 USA; 4 Department of Forest and Rangeland Stewardship, Colorado State University, Fort Collins, CO 80523 USA

**Keywords:** Allometric equations, Carbon, Landsat, *Pseudotsuga menziesii*, *Pinus contorta*, *Pinus ponderosa*, Uncertainty

## Abstract

**Background:**

Biomass maps are valuable tools for estimating forest carbon and forest planning. Individual-tree biomass estimates made using allometric equations are the foundation for these maps, yet the potentially-high uncertainty and bias associated with individual-tree estimates is commonly ignored in biomass map error. We developed allometric equations for lodgepole pine (*Pinus contorta)*, ponderosa pine (*P. ponderosa)*, and Douglas-fir (*Pseudotsuga menziesii)* in northern Colorado. Plot-level biomass estimates were combined with Landsat imagery and geomorphometric and climate layers to map aboveground tree biomass. We compared biomass estimates for individual trees, plots, and at the landscape-scale using our locally-developed allometric equations, nationwide equations applied across the U.S., and the Forest Inventory and Analysis Component Ratio Method (FIA-CRM). Total biomass map uncertainty was calculated by propagating errors from allometric equations and remote sensing model predictions. Two evaluation methods for the allometric equations were compared in the error propagation—errors calculated from the equation fit (equation-derived) and errors from an independent dataset of destructively-sampled trees (n = 285).

**Results:**

Tree-scale error and bias of allometric equations varied dramatically between species, but local equations were generally most accurate. Depending on allometric equation and evaluation method, allometric uncertainty contributed 30–75% of total uncertainty, while remote sensing model prediction uncertainty contributed 25–70%. When using equation-derived allometric error, local equations had the lowest total uncertainty (root mean square error percent of the mean [% RMSE] = 50%). This is likely due to low-sample size (10–20 trees sampled per species) allometric equations and evaluation not representing true variability in tree growth forms. When independently evaluated, allometric uncertainty outsized remote sensing model prediction uncertainty. Biomass across the 1.56 million ha study area and uncertainties were similar for local (2.1 billion Mg; % RMSE = 97%) and nationwide (2.2 billion Mg;  % RMSE = 94%) equations, while FIA-CRM estimates were lower and more uncertain (1.5 billion Mg;  % RMSE = 165%).

**Conclusions:**

Allometric equations should be selected carefully since they drive substantial differences in bias and uncertainty. Biomass quantification efforts should consider contributions of allometric uncertainty to total uncertainty, at a minimum, and independently evaluate allometric equations when suitable data are available.

## Background

Spatially explicit aboveground biomass estimates are critical for monitoring forest carbon storage and for strategic forest planning [1–3]. They provide baseline inventories that capture the legacy of past land use and disturbance while also serving as a reference point for studying the impacts of subsequent disturbances [4]. Forest biomass maps are also a critical tool for measuring, reporting, and verifying forest carbon stocks [5]. Programs such as Reducing Emissions from Deforestation and forest Degradation (REDD+) and California cap-and-trade seek to mitigate rising greenhouse gas concentrations by storing carbon in forests. The financial incentives tied to forest carbon in these programs have lead countries and forest landowners to closely track their forest carbon. Individual-tree biomass calculated from allometric equations are the foundation for these estimates, but can have high uncertainty and bias that propagate to biomass/carbon estimates [6, 7]. Widely-used allometric equations must be independently evaluated using tree biomass datasets to identify error and bias [8].

Allometric equations provide biomass estimates from tree measurements such as diameter at breast height (DBH), height, and/or wood density. These equations capture the scaling relationships between tree form and function to predict total and component (e.g., branch, needle, bark, bole, root) biomass [9]. Allometric relationships are commonly developed from trees sampled across large areas [10–12]. In the United States (U.S.), two widely-applied allometric equations are the Forest Inventory and Analysis Component Ratio Method (FIA-CRM) [13, 14] and Jenkins et al. [11]. The FIA-CRM is used to calculate forest carbon for the U.S. Environmental Protection Agency’s (EPA) annual greenhouse gas inventory [15] and for California cap-and-trade projects. The Jenkins et al. [11] equations were used for the EPA annual greenhouse gas inventory in the past, are part of the FIA-CRM, and are an option in the Fire and Fuels Extension of the Forest Vegetation Simulator [16].

Allometric equations are frequently applied outside populations from which they were developed, potentially leading to significant biomass estimation errors [17]. Allometric relationships vary spatially with differences among trees (i.e., species and genetics) and growing conditions (i.e., site productivity arising from nutrient availability, soil type, and climate, competition and tree age) [18–20]. Where available, locally-developed equations offer an alternative to more generic equations and can be better tuned to local species, growth forms, and environments. However, locally-developed allometric equations are typically developed from small sample sizes, potentially rendering them biased, unreliable, and prone to measurement error [7, 17]. Some studies suggest locally-developed allometric equations are more accurate [21–23], while other studies have found that generic allometric equations perform better [18, 24, 25]. Another method for developing more robust allometric equations is to tune generic allometric equations to better represent local growth forms. For example, the FIA-CRM uses regional stem volume equations to rescale biomass predictions made by generic allometric equations [13]. Ultimately, those conducting biomass inventories are left with the choice of selecting allometric equations, or must take on the time-consuming, expensive, and difficult task of building their own allometric equations. The choice of allometric equation can have significant impacts on biomass estimates [22, 26].

To map biomass across landscapes, allometric biomass equations are applied to individual tree measurements, which are summed across forest inventory plots that calibrate larger-scale remote sensing datasets (e.g., [26]). Biomass and forest structure are frequently mapped using freely-available images from Landsat satellites [27–30]. Landsat satellites acquire moderate resolution (30 m × 30 m pixel size) multispectral data that have an extensive historical record spanning 1972 to present, making these sensors ideal candidates for ecological monitoring and estimating forest productivity [31]. Landsat spectral bands, vegetation indices, and texture metrics are useful predictors of forest biomass [27, 32–35]. Since Landsat and other optical sensors rely on detectable changes in canopy closure, one issue is underestimation, or saturation, of predictions at high biomass values and in closed-canopy forests. Landsat-based biomass mapping may, however, be aided in the western U.S. by the strong biophysical gradients of forest type and biomass and the open canopies of some of the forests [28]. Biomass predictions can be improved by supplementing remote sensing imagery with other data sources to capture correlations between forest biomass and climate, topography, and landform [36]. Active sensors, such as LiDAR (Light Detection and Ranging), can also improve Landsat-based biomass estimates by providing accurate information about forest structure and height [37], but spatial and temporal data coverage is limited and collection is expensive.

There are many sources of uncertainty when mapping biomass—tree measurements, allometric models, plot representativeness, and remote sensing model fitting and prediction [7, 38]. Biomass maps are commonly evaluated by comparing predicted pixel biomass to observed plot biomass values, treating the plot biomass as “truth.” This approach only captures one source of error: variability or errors in the remote sensing model. While this is certainly a major error source, failing to propagate other error sources underlying tree and thus plot-level biomass calculations under-estimates uncertainty [6, 38]. Allometric model uncertainty has been found to account for the majority of the tree-level uncertainty, and can be biased [7, 39]. Tree measurement errors of attributes such as DBH and height can also be significant [38]. Since allometric error is generally calculated from the same trees used to develop the equations (i.e. lacking independent validation), issues such as sampling bias may not be captured in uncertainty measures. Although it is rare to have an independent dataset of destructively sampled trees, allometric error and bias are best captured by comparing predictions to trees within the study area that are independent of the allometric equation generation.

In this study, we estimate standing aboveground forest biomass using multiple allometric equations for montane and subalpine forests in the southern Rocky Mountains. We develop local allometric biomass equations for lodgepole pine (*Pinus contorta)*, ponderosa pine (*P. ponderosa*), and Douglas-fir (*Pseudotsuga menziesii*) and use them to estimate biomass in Forest Inventory and Analysis (FIA) plots. With these plot-level biomass estimates, Landsat Enhanced Thematic Mapper Plus (ETM+) imagery, and geomorphometric and climate layers we use a machine learning algorithm to calibrate several biomass maps covering 1.56 million ha. We analyze the magnitude and patterns of biomass differences at the tree, plot, and landscape scale between locally-developed allometric equations and two widely-used allometric biomass equations: Jenkins et al. [11] and the FIA-CRM [13, 14]. Using an independent validation dataset [40], we evaluate accuracy and bias of the three allometries. Finally, we propagate allometric error to the final remote sensing calibration model to quantify biomass map uncertainties and the relative importance each error source.

## Methods

### Study area

**Fig. 1:**
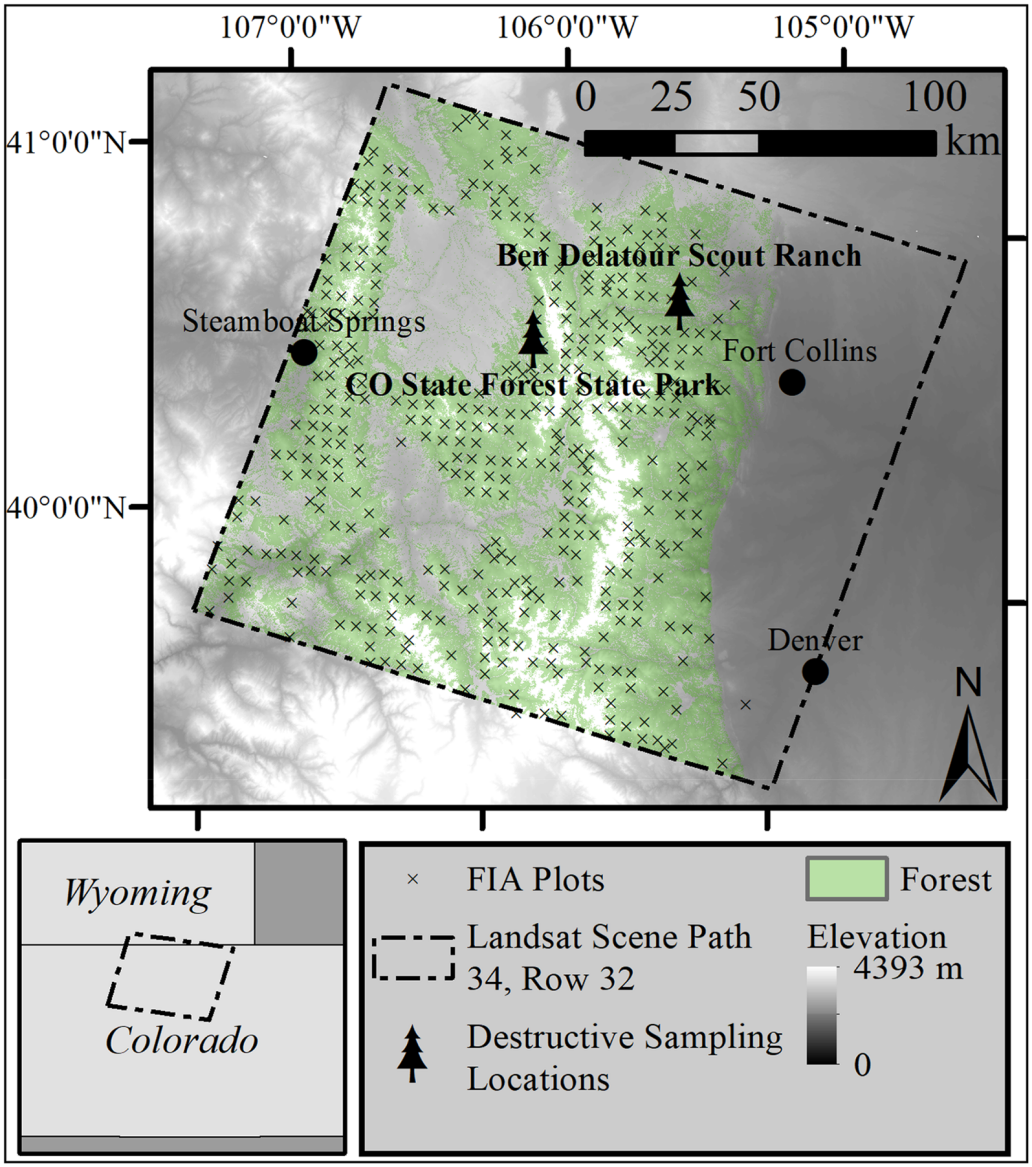
Study area map showing the destructive sampling sites, the approximate locations of Forest Inventory and Analysis (FIA) plots, and the forest extent within Landsat scene path 34, row 32

This study was conducted across the 1.56 million ha of forest in northern Colorado and southern Wyoming bound by Landsat scene path 34, row 32 (Fig. 1). Mean temperature and precipitation vary along an elevation gradient from 52 °C and 31 cm at lower elevation montane forests to − 3 °C and 180 cm at higher elevations subalpine forests [41]. Forest species composition also changes with elevation and aspect. Montane forests dominated by ponderosa pine start between 1540 m and 1845 m above sea level (asl) and become more mixed with Douglas-fir and quaking aspen (*Populus tremuloides*) as elevation increases [42]. Douglas-fir is particularly common on north-facing slopes. Lodgepole pine and limber pine (*Pinus flexilis*) join the species mix at about 2460 m asl. Lodgepole pine is the dominant tree species above 2770 m, mixed with quaking aspen, limber pine, subalpine fir (*Abies lasiocarpa*) and Engelmann spruce (*Picea engelmannii*). These lodgepole pine forests experienced extensive mountain pine beetle (*Dendroctonus ponderosae* Hopkins) induced tree mortality starting at low levels in the early 2000s, peaking between 2006 and 2009, and declining in 2010 [43]. Subalpine fir and Engelmann spruce take over as the predominant tree species between 3077 m and treeline (~ 3540 m asl) [42].

### Allometric equations

#### Destructive sampling

The destructive biomass sampling required to build the local allometric biomass equations was conducted at two sites (Fig. 1). Lodgepole pine was sampled at the Colorado State Forest and the ponderosa pine and Douglas-fir trees were sampled at the Ben Delatour Boy Scout Ranch. We selected trees free of deformities that represented the diameter range in each area: 20 lodgepole pine, 10 ponderosa pine, and 10 Douglas-fir. The larger-diameter lodgepole pine (n = 14) were sampled in a mature, even-aged stand at 2700 m asl that was impacted by a mountain pine beetle outbreak around 2007 which killed 75% of the basal area, as measured by inventory plots around the destructively sampled trees. The smaller trees (< 14 cm; n = 6) were sampled near the mature lodgepole pine sampling site in a regenerating clearcut at 2800 m asl that was pre-commercially thinned a year prior to destructive sampling. Both stands were lodgepole pine-dominated. The ponderosa pine trees were sampled from a mixed-age forest at 2300 m asl that had irregular structure consisting of patches of trees as well as open-grown trees. While ponderosa pine was the dominant tree in this area, Rocky Mountain juniper (*Juniperus scopulorum*) were interspersed. The Douglas-fir were sampled from a Douglas-fir-dominated stand at 2300 m asl that also contained ponderosa pine and Rocky Mountain juniper.

The destructive sampling procedure [44, 45] was designed to measure dry biomass of the bole, bark, branch, and foliage components, as well as total aboveground biomass of each tree. The bole is the main stem (without bark) between the 1-foot stump and where the bole reaches a 10.2 cm (4 inch) diameter, hereafter referred to as the 10.2 cm top. The bark component is all bark on this same portion of the main stem. Foliage is all foliage on the tree. Branch biomass includes wood and bark of the main stem above the 10.2 cm top and all other branches. In our destructive sampling procedure, the wet mass of the whole tree was weighed as components in the field and then subsamples were retained to determine moisture contents. These methods are described in detail in Stovall et al. [44] and Additional file 1. Methods for calculating the biomass of each component are also detailed in Additional file 1.

#### Allometric equation calculation

Allometric biomass equations for the components of each tree were generated using nonlinear seemingly unrelated regression. Seemingly unrelated regression is well-suited for allometric biomass equations because it allows for dependencies between error terms of the component equations and the equations can be constrained to ensure tree components sum to total aboveground biomass [45–48]. We used the logarithmic model form,

1 $$Biomass = { \exp }\left( {a_{11} + a_{12} *\ln \left( {DBH} \right)} \right)$$

which is commonly used for tree biomass estimation [11, 47, 49] and appeared to fit scatterplots of our biomass data for all three species. We implemented the nonlinear model form rather than the log–log transformed linear model to avoid back-transformations and to allow for the inclusion of component zero values for trees too small to have bole and bark biomass under our component definitions. Nonlinear seemingly unrelated regression was implemented in SAS OnDemand software [50] to estimate parameters for the following set of equations for lodgepole pine, ponderosa pine, and Douglas-fir:

2 $$Bole = { \exp }\left( {a_{11} + a_{12} X} \right)$$

3 $$Bark = { \exp }\left( {a_{21} + a_{22} X} \right)$$

4 $$Foliage = { \exp }\left( {a_{31} + a_{32} X} \right)$$

5 $$Branch = { \exp }\left( {a_{41} + a_{42} X} \right)$$

6 $$Total = \exp \left( {a_{11} + a_{12} X} \right) + e{\text{xp}}\left( {a_{21} + a_{22} X} \right) + \exp \left( {a_{31} + a_{32} X} \right) + {\text{exp}}\left( {a_{41} + a_{42} X} \right)$$

where a_ij_ are the regression parameters (j = 1, 2) to be estimated for each component (i = 1, 2, 3, 4) and X is the natural logarithm of DBH in cm. Biomass values are in kg. Parameter start values were estimated by solving the linear version of each component model using ordinary least squares [46].

#### Allometric equation comparison

We estimate aboveground biomass for trees in FIA plots within the study area using three sets of allometric equations. The three sets of allometric equations tested are: (1) the equations presented in Jenkins et al. [11], (2) the FIA-CRM and (3) a local set of equations. The allometric biomass equations presented by Jenkins et al. [11] for U.S. tree species were developed using pseudodata generated from published allometric equations. These equations predict total aboveground biomass and component biomass (i.e., foliage, coarse roots, stem bark, stem wood, and branches) as a proportion of aboveground biomass for species groups (e.g., pine, spruce, true fir/hemlock, etc.) from tree diameter at breast height. The FIA-CRM method estimates biomass using a fusion of Jenkins et al. [11] equations and regional stem volume equations compiled in Woodall et al. [14]. Regional equations are used to estimate volume of the merchantable stem, which is then converted to biomass using specific gravity values found in Miles and Smith [51]. Volume estimates account for species, diameter, height, and atypical tree form to deduct missing or rotten bole mass [13]. The FIA-CRM equations do not estimate foliage biomass. Additional information about how biomass is calculated using FIA-CRM can be found in Additional file 1.

Local biomass estimates were made from a variety of allometric equations depending on species. As described above, we developed equations for three dominant tree species in our study area: lodgepole pine, ponderosa pine, and Douglas-fir. For other species, we used equations from the literature that were developed as near as we could find to the study area. We applied equations from Landis and Mogren [52] for Engelmann spruce and Johnston and Bartos [53] for aspen. Species covered by the allometry presented in this paper and local equations from the literature accounted for 77% of the basal area in the FIA plots used in this study. We estimated biomass for all other species (23% of plot basal area) using the FIA-CRM biomass since the FIA-CRM estimates are designed to be more tuned to local conditions.

### Biomass estimation

#### Forest inventory data

We used FIA plot data to evaluate biomass variability between allometric equations at the plot scale and to map standing aboveground tree biomass. The FIA program is responsible for systematically monitoring U.S. forests on a 5–10 year cycle [54]. Each FIA plot consists of four 7.3 m radius circular subplots where trees with a diameter at breast height of 12.70 cm or greater are measured. Saplings, defined as having a diameter at breast height between 2.54 and 12.70 cm, are measured in 2.1 m radius microplots nested within the subplots. Sapling biomass was included in this analysis, but biomass of trees less than 2.54 cm diameter was excluded. We calculated and compared standing aboveground tree biomass at FIA plots measured between 2002 and 2015 (n = 418) using Jenkins et al. [11], FIA-CRM and the local set of allometric equations. FIA remeasures plots in Colorado about every 10 years—only data from the measurement closest to the satellite imagery capture date (2001) were used. We also only used plots designated as a single condition (i.e., forest or land cover type) to avoid spectral confusion that could be caused by heterogeneous plots [55].

Plots sampled between 2002 and 2015 and satellite imagery from 2001 were used to map total aboveground standing biomass before the mountain pine beetle epidemic caused widespread lodgepole pine mortality in our study area. Plot measurements captured tree mortality not reflected by the 2001 imagery, especially for plots sampled at the latter end of this sampling timeframe. We mitigated differences between image and sampling conditions by including all standing trees, both living and dead, in plot biomass estimates. While some localized areas may have had significant treefall by 2015, a study in this same area found no change in downed woody material 7 years after this outbreak [56]. Most plots were measured towards the beginning of this sample period when dead trees were likely to still be standing. Between 35 and 46 plots were sampled annually between 2002 and 2011 for a total of 393 plots, while only 25 total plots were sampled between 2012 and 2015. Another discrepancy between plot data and the imagery is that the plots continued to grow between image capture and the date-of-sampling. This discrepancy was minor considering the relatively slow growth in this region (e.g., average of 0.94 cm decade^−1^ diameter increment for lodgepole pine) [Bagdon, B., Nguyen, T., Vorster, A.G., Paustian, K., Field, J., unpublished observations] and the other, larger sources of uncertainty. Errors resulting from the temporal mismatch between imagery and plot measurement were deemed worth the tradeoff for more plots to train the remote sensing models.

#### Mapping Aboveground Forest Biomass

We used a combination of geomorphometric, topographic, climatic, and spectral predictor variable layers to map biomass. Spectral bands, vegetation indices, and image textures were all generated from a Level 1 Terrain-corrected (L1T) Landsat 7 ETM + image captured on September 24, 2001. The image was accurately geometrically registered (Root Mean Square Error [RMSE] = 3.2 m). Areas flagged by the C Function of Mask (CFMask) algorithm as water, cloud shadow, snow, or cloud overlapped with 0.04% of the forested area and were removed from the study. We used digital number for the ETM + bands, texture, and most vegetation indices since the study encompassed a single scene and point in time [57]. Some vegetation indices required top-of-atmosphere reflectance (second modified soil-adjusted vegetation index, Tasseled Caps, soil-adjusted vegetation index) or surface reflectance (enhanced vegetation index). For these indices, the top-of-atmosphere reflectance and Landsat Climate Data Record surface reflectance products were used. We used the least processed imagery necessary for each predictor variable [57]. In addition to ETM + bands, vegetation indices, and texture [27, 33–35], we also generated topographic, geomorphometric, and climatic predictor variables that have been shown to correlate with tree species and biomass distributions [36, 58]. See Additional file 1 for more information about how these predictor variables were generated and Additional file 1: Table S4 for a list of all predictor variables generated. We had 302 total predictor variables between all band (n = 7), index (n = 16), texture (n = 240), climate (n = 16), and topography and geomorphology layers (n = 23). Values of each predictor variable were extracted for FIA plots using a 3 × 3 pixel mean since the four FIA subplots cover an area roughly this size [55].

We mapped biomass only within forested pixels in the study area, as defined by a forest mask developed for this area [59]. This mask only includes pixels with greater than or equal to ten percent canopy cover as defined by the LANDFIRE Existing Vegetation Cover [60] product. This aligns with the ten percent canopy cover requirement component of the FIA forest definition.

We mapped standing aboveground tree biomass using a random forest model which is commonly used for remote sensing applications and biomass mapping [28, 61, 62]. Random forest models are efficient, non-parametric, have strong prediction accuracy, can handle large numbers of predictor variables, and are robust to noise and outliers [63]. In this approach, a regression tree is trained with a random subset of training data and with a random selection of predictor variables at each node. This process is repeated many times to build a “forest” of unpruned decision trees. Predictions are made as the average of the predictions from all trees [64]. Users define the number of trees (ntree) and number of parameters considered at each node (mtry), although the models are relatively stable to parameter adjustments. Data withheld from each tree (out-of-bag data) are used to calculate reliable estimates of error and variable importance, reducing the need to withhold test data [63]. We evaluate the random forest models using pseudo R^2^, RMSE, and bias. These three evaluation metrics are indicative of model fit: pseudo R^2^ indicates the proportion of the variability explained by the model, RMSE reflects the magnitude of errors between predicted and observed values, and bias shows the degree to which models tend to over- (positive bias) or under-predict (negative bias). Bias and RMSE are also reported as percentages of the observed mean.

We had a large number of predictor variables, so we implemented a data-driven variable selection technique suitable for applications such as this study where prediction is the goal (Additional file 1: Table S5) [65]. This method, Variable Selection Using Random Forest (VSURF) [65], is described in Additional file 1. After implementing VSURF, we removed variables from variable pairs correlated by 0.7 or more, keeping the variable with the higher variable importance from a random forest model run with all variables in the VSURF prediction set. We measured predictor variable importance by the average decrease in the mean squared error attributable to a particular variable across all trees [64]. Several variables correlated by up to 0.75 were retained if the correlated variables contained unique information and retaining both improved model performance.

We repeated this variable selection routine for each set of biomass values (local, Jenkins et al. [11] and FIA-CRM) and used the selected predictor variables in the randomForest package [64]. We tested a range of mtry (1-# of predictors) and ntree (500, 1000, 1500, 2000, 3000, 4000, 5000) values in 10 iterations and selected the parameters that most frequently lead to the smallest out-of-bag errors.

### Biomass variability and uncertainty

#### Biomass variability across tree, plot, and landscape scales

At the tree, plot, and landscape scales, we compared the magnitude and patterns of differences in biomass estimates between the three sets of allometric equations. At the tree scale, component biomass and total biomass excluding foliage were compared for the species we destructively sampled: lodgepole pine, ponderosa pine, and Douglas-fir. Only total biomass excluding foliage was compared for Engelmann spruce, subalpine fir, and aspen. At the plot and landscape scale, we compared aboveground biomass for all species.

Some component definitions differed between the three sets of allometric equations so we made the necessary adjustments. Jenkins et al. [11] and local equations estimated biomass for the same components: bole, bark, branch, and foliage. When comparing local to Jenkins et al. [11] component biomass estimates, the only adjustment needed was to subtract stump biomass as calculated by FIA-CRM [66] from the Jenkins et al. [11] branch biomass estimates. This branch component aligned with the FIA-CRM branch biomass definition (sometimes called the top component). The FIA-CRM bole component spanned the same portion of the tree as our local and Jenkins equations but included the bark. So, bole and bark together were compared across all three sets of allometric equations at the tree scale. Another difference in components between estimation methods is that FIA-CRM does not calculate foliage biomass. For tree-scale total biomass comparisons, foliage biomass is excluded from both local biomass estimates and Jenkins et al. [11] estimates. However, foliage is included for local and Jenkins et al. [11] estimates, but not FIA-CRM estimates, in plot and landscape-scale comparisons. For the subset of tree species where local biomass estimates were generated from FIA-CRM equations, foliage biomass as estimated by Jenkins et al. [11] was added to each tree for plot and landscape-scale local biomass estimates. Aboveground biomass included the stump for FIA-CRM and Jenkins et al. [11] estimates, but not for our local equations. We remedied this by adding stump biomass as estimated by FIA-CRM [66] to each tree when calculating local total biomass for tree, plot, and landscape scale analyses.

At all scales (tree, plot, and landscape), we measured variability between biomass estimates by calculating the mean difference and mean relative difference. The difference between each set of allometric equations was calculated at each scale: (1) local—Jenkins et al. [11], (2) local—FIA-CRM, and (3) Jenkins et al. [11]—FIA-CRM. We calculated relative differences by dividing differences by the minuend. We analyzed tree, plot, and landscape scale differences by calculating the mean and relative differences across all trees in the FIA plots, FIA plots (i.e., the sum of trees within each plot), and pixels. To better understand patterns in variability between allometric equations at the tree scale, we calculated biomass differences for each species we sampled (lodgepole pine, ponderosa pine, and Douglas-fir) for each component and in 20 cm diameter bins.

At the plot scale, we identified the stand characteristics most correlated with biomass estimate differences between different sets of allometric equations. This was done using a random forest model to predict the plot biomass difference between allometric equations using stand structure and composition predictor variables. Details about this analysis can be found in Additional file 1. At the landscape scale, we evaluated patterns in allometric equation differences by summarizing biomass differences and relative differences by forest type in 2001 as defined by LANDFIRE version 1.0.5 Existing Vegetation Type [67].

#### Allometric error

Allometric biomass error is not reported in some cases (e.g., FIA-CRM) and, when it is reported, the error is simply the error or variability in the model fit (e.g., Jenkins et al. [11] and our local equations). To better understand the representativeness of allometric equations in our study area, we evaluated all three equations using an independent dataset of destructively sampled lodgepole pine, ponderosa pine, and Douglas-fir trees from the Legacy Tree Database [40]. Calculating precision and bias with an independent set of trees allowed us to compare allometric model performance in our study region, while also enabling error estimates for the FIA-CRM predictions.

We used 285 lodgepole pine, ponderosa pine, and Douglas-fir trees from the Legacy Tree Database to calculate allometric errors (Additional file 1: Table S6). Trees located in Colorado and Wyoming were considered, although no trees from Wyoming fit our criteria. We used dry weights of all above-stump bark and wood for 73 lodgepole and ponderosa pine trees from near Red Feather Lakes in northern Colorado [68, 69] which is within the study area and is near our ponderosa pine and Douglas-fir sampling sites (Table 1). Reid [68] destructively sampled 19 lodgepole pine trees at around 3000 m elevation. Tossey [69] sampled seedlings and saplings across a range of site qualities, topographic positions, and habitat types between elevations of 1700 m and 3700 m. The other 212 Legacy trees used were sampled in National Forests within and just outside of the study area, but only green mass was reported [70]. For these trees, we converted above-stump green mass to dry mass using the steps described in Additional file 1. Comparing biotic and abiotic growth conditions (e.g., trees ha^−1^, basal area, site index, precipitation) between our sites and Legacy Tree sites could help explain differences between biomass observations and predictions, but comparable information was not available across studies.

**Table 1:** Destructively sampled trees used in this study (Legacy Trees and trees destructively sampled for this study), the number of trees sampled for each species, location, and study and a summary of the diameter at breast height (DBH)

Species	Study	Location	n	Mean DBH (cm)	Min DBH (cm)	Max DBH (cm)
Lodgepole pine	This study	CO State Forest	20	16.3	2.5	29.9
	Reid et al. (1974)	Near Red Feather Lakes, CO	19	13.3	2.5	28.7
	Sánchez Meador (2007)	Pike, San Isabel, and Aarapaho NF	69	13.2	1.5	32.0
	Tossey (1982)	Near Red Feather Lakes, CO	26	5.4	1.0	11.4
Ponderosa pine	This study	Ben Delatour Boy Scout Ranch	10	34.0	4.9	61.8
	Sánchez Meador (2007)	Pike, San Isabel, and Aarapaho NF	80	15.3	1.8	36.6
	Tossey (1982)	Near Red Feather Lakes, CO	28	5.3	0.8	11.7
Douglas-fir	This study	Ben Delatour Boy Scout Ranch	10	24.9	2.4	46.6
	Sánchez Meador (2007)	Pike, San Isabel, and Aarapaho NF	63	14.1	1.5	39.6

*NF* National Forest, *CO* Colorado

For each Legacy tree, we estimated biomass using each set of allometric equations. We adjusted local, Jenkins et al. [11], and FIA-CRM estimates to match components measured for the Legacy trees by subtracting foliage mass from local and Jenkins et al. [11] estimates and by subtracting stump mass from Jenkins et al. [11] and FIA-CRM estimates. For FIA-CRM, we did not have all information needed to calculate biomass of Legacy trees so we employed an alternative method. FIA-CRM biomass was estimated as the biomass of the tree in the FIA plot data most similar to the Legacy tree. We matched Legacy trees to a tree in the FIA plot data by extracting the 20 FIA trees most similar in diameter at breast height to the Legacy tree and then selecting the tree closest in height to the Legacy tree from this list of 20. The diameter and height of trees for which the FIA-CRM biomass values were used matched Legacy trees within 0.1 cm diameter at breast height on average (sd = 0.6 cm) and < 0.1 m height (sd = 0.5 m). We report relative and absolute RMSE and bias between Legacy tree biomass and predictions from each set of allometric equations.

#### Uncertainty propagation

We propagated error from two important contributors to total biomass prediction uncertainty [7, 38, 39]: error from allometric biomass equations (hereafter “allometric error”) and from the remote sensing model predictions used to map biomass (hereafter “prediction error”). We calculated uncertainty at the tree, plot, and pixel scale and compared the relative contributions of allometric error and prediction error using methods from Chen et al. [38] and Stovall and Shugart [39]. We do not account for errors in the predictor variable measurement (i.e., DBH or spectral, topographic, and climatic layers) or model parameters of the allometric and remote sensing models and, thus, underestimate total uncertainty. Past work has highlighted allometric and prediction errors as the primary sources of biomass prediction error [38]. Moreover, our primary goal was to better understand the relative contribution from each of these error sources, as opposed to quantifying total uncertainty in each scenario. We hypothesize adding the additional sources of error would scale our overall results, increasing the total amount of uncertainty, but relative contributions from allometry and remote sensing model-based predictions would likely remain similar.

**Fig. 2:**
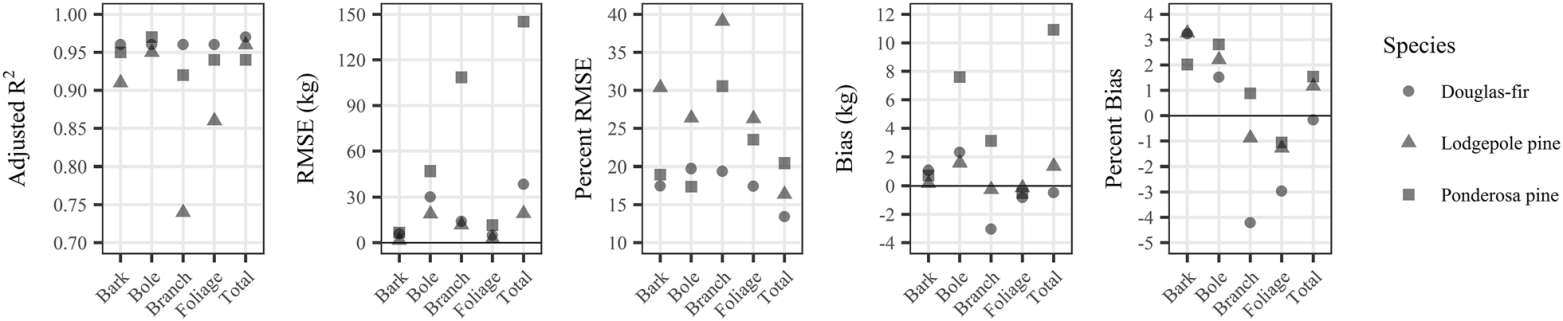
Evaluation metrics for allometric equations generated in this study for each component and species

We propagated allometric error from two scenarios—one with the errors from the equation fit (equation-derived) and one using errors calculated from comparisons with the independent Legacy Tree Data. Both error scenarios were propagated for local and Jenkins et al. [11] allometric equations, but we only propagate Legacy Tree Data allometric error for FIA-CRM since error is not reported for FIA-CRM equations. We only had error for our three focal species when propagating allometric error from Legacy Tree Data evaluations, so errors reported in Jenkins et al. [11] were used for all other species across all three sets of allometric equations. For equation-derived evaluation of local biomass estimates, allometric error ($\sigma_{tree}$) for our three focal species was the total tree biomass relative RMSE from our equations (Fig. 2). We used the standard error reported in Landis and Mogren [52] for Engelmann spruce in the local equation-derived evaluation. The aspen biomass equations used in our local estimates [53] only reported R^2^, so we utilized Jenkins et al. [11] uncertainty for these trees and for all other species. Jenkins et al. [11] errors are reported as RMSE in natural log units. We calculated Jenkins et al. [11] allometric error ($\sigma_{tree}$, kg) for each tree using the following equation.

7 $$\sigma_{tree} = \frac{{e^{{(\beta_{0} + \beta_{1} \ln DBH) + 1.96*RMSE}} - e^{{(\beta_{0} + \beta_{1} \ln DBH) - 1.96*RMSE}} }}{2*1.96}$$

where $\beta_{0}$, $\beta_{1}$, and RMSE are species group-specific regression parameters and errors from Table 4 of Jenkins et al. [11]. Allometric uncertainty is propagated to the plot level ($\sigma_{plot}$, Mg ha^−1^) using the following equation [38, 39]

8 $$\sigma_{plot} = \sqrt {\mathop \sum \limits_{i = 1}^{{n_{tree, plot} }} \frac{{\sigma_{tree,i}^{2} }}{s}}$$

where $s$ is the area of the plot in hectares.

This plot-level allometric uncertainty was combined and propagated with remote sensing model prediction error ($\sigma_{{\varepsilon ,\hat{B}_{plot} }}$) using the following equation. The model prediction error was the RMSE for the random forests model built with the respective set of allometric biomass equations.

9 $$\sigma_{pred} = \sqrt {\sigma_{\varepsilon ,plot}^{2} + \sigma_{{\varepsilon ,\hat{B}_{plot} }}^{2} }$$

where $\sigma_{pred}$ is the total uncertainty from allometric and prediction error in Mg ha^−1^. We calculate percent uncertainty by dividing by the mean plot-level biomass density and evaluate the relative contribution of each error source as the percentage of $\sigma_{pred}$.

## Results

### Destructive sampling and local allometric equations

Destructively sampled trees spanned the diameter range observed at each site (Table 2). The lodgepole pine tended to have the smallest DBH and grow at the highest density, while the ponderosa pine had the largest DBH and grew in the lowest densities. We calculated dry total and component biomass for each destructively sampled tree. The multiple regression equations (Additional file 1: Table S2) developed for predicting single branch length, foliage mass, and wood mass performed strongly, with adjusted R^2^ values averaging 0.84 and ranging from 0.63 to 0.98 (Additional file 1: Table S3). Predictions from these equations were used in combination with other in-field measurements to calculate component biomass (Table 2). For all species, the majority of aboveground biomass was in the bole and branch components, and trees had more foliage than bark biomass (Additional file 2: Fig. S1). Moisture content for each component and species are presented in Additional file 2: Table S1. Specific gravity of the bole increased from lodgepole pine (0.39) to ponderosa pine (0.42) to Douglas-fir (0.43). Table 3 presents regression coefficients for Eqs. (2–6) predicting component biomass from a tree’s diameter at breast height for each species. Parameter estimates were stable to variations in their start values. Allometric equations fit the data well, with all but two components having adjusted R^2^ values greater than 0.90 (Fig. 2). Absolute RMSE values were highest for ponderosa pine, but lodgepole pine tended to have the highest relative errors (Fig. 2). Across species, allometric equations over-estimated bark and bole biomass, but under-estimated foliage biomass (Fig. 2).

**Table 2:** Height, diameter at breast height (DBH), and stand structure of destructively sampled trees and from 7.32 m radius plots measured around each tree and total and component biomass of the destructively sampled trees

	Species	Mean	Standard deviation	Min	Max
DBH (cm)	PSME	24.9	15.4	2.4	46.6
	PICO	16.3	8.0	2.5	29.9
	PIPO	34.0	18.5	4.9	61.8
Height (m)	PSME	12.7	5.3	3.2	19.7
	PICO	12.2	6.5	3.2	21.1
	PIPO	11.3	4.3	3.0	16.8
Tree density (trees ha^−1^)	PSME	386	176	119	535
	PICO	921	414	238	2139
	PIPO	172	162	0	416
Basal area (m^2^ ha^−1^)	PSME	15.6	6.5	7.2	30.3
	PICO	18.6	14.9	0.5	44.9
	PIPO	13.4	10.4	0	29.7
Average plot DBH (cm)	PSME	21.2	7.4	12.2	35.9
	PICO	15.3	7.2	4.9	27.0
	PIPO	26.6	17.6	0.0	65.0
Total biomass^a^ (kg)	PSME	286.3	290.1	3.2	809.1
	PICO	117.6	114.7	1.7	358.4
	PIPO	710.4	751.7	5.7	2188.1
Stem wood biomass^b^ (kg)	PSME	152.6	166.1	0	434.2
	PICO	72.1	83.5	0	247.2
	PIPO	270.4	268.1	0	738.5
Stem bark biomass (kg)	PSME	33.1	31.3	0	81.8
	PICO	5.3	5.6	0	15.8
	PIPO	35.5	32.3	0	89.7
Foliage biomass (kg)	PSME	28.2	25.4	1.2	72.1
	PICO	10.2	7.3	0.8	24.1
	PIPO	49.2	49.9	1.5	153.9
Branch biomass (kg)	PSME	72.4	70.5	2.0	220.9
	PICO	30.1	23.7	0.9	82.0
	PIPO	355.3	410.8	4.2	1206.0

*PSME* Douglas fir (*Pseudotsuga menziesii;* n = 10*)*, *PICO* lodgepole pine (*Pinus contorta;* n = 20), *PIPO* ponderosa pine (*P. ponderosa;* n = 10)

^a^Includes foliage

^b^Stem wood does not include bark

**Table 3:** Allometric biomass equation regression coefficients for Eqs. (2–6) for lodgepole pine, ponderosa pine, and Douglas-fir

Species	Component	a_i1_	a_i2_
Douglas-Fir	Bole (i = 1)	− 2.9162 (0.9896)	2.3437 (0.2647)
	Bark (i = 2)	− 2.0888 (0.7021)	1.6911 (0.1903)
	Foliage (i = 3)	− 3.3489 (0.8356)	1.9822 (0.2249)
	Branch (i = 4)	− 3.7741 (1.0634)	2.3588 (0.2845)
Lodgepole Pine	Bole (i = 1)	− 4.3642 (0.8611)	2.9255 (0.2634)
	Bark (i = 2)	− 5.2333 (0.8573)	2.3723 (0.2646)
	Foliage (i = 3)	− 2.0830 (0.6570)	1.5402 (0.2075)
	Branch (i = 4)	− 1.0172 (0.9174)	1.5475 (0.2897)
Ponderosa Pine	Bole (i = 1)	− 2.5513 (0.8855)	2.2322 (0.2231)
	Bark (i = 2)	− 3.5399 (0.9608)	1.9588 (0.2432)
	Foliage (i = 3)	− 5.75806 (1.2645)	2.6110 (0.3168)
	Branch (i = 4)	− 5.2127 (1.6641)	2.9843 (0.4149)

Equations were fit using nonlinear seemingly unrelated regression to estimate component biomass (kg) from a tree’s diameter at breast height (cm). Values in parentheses are standard errors of the parameter values. Total biomass can be calculated as the sum of these four components

### Tree-scale biomass variability

**Fig. 3:**
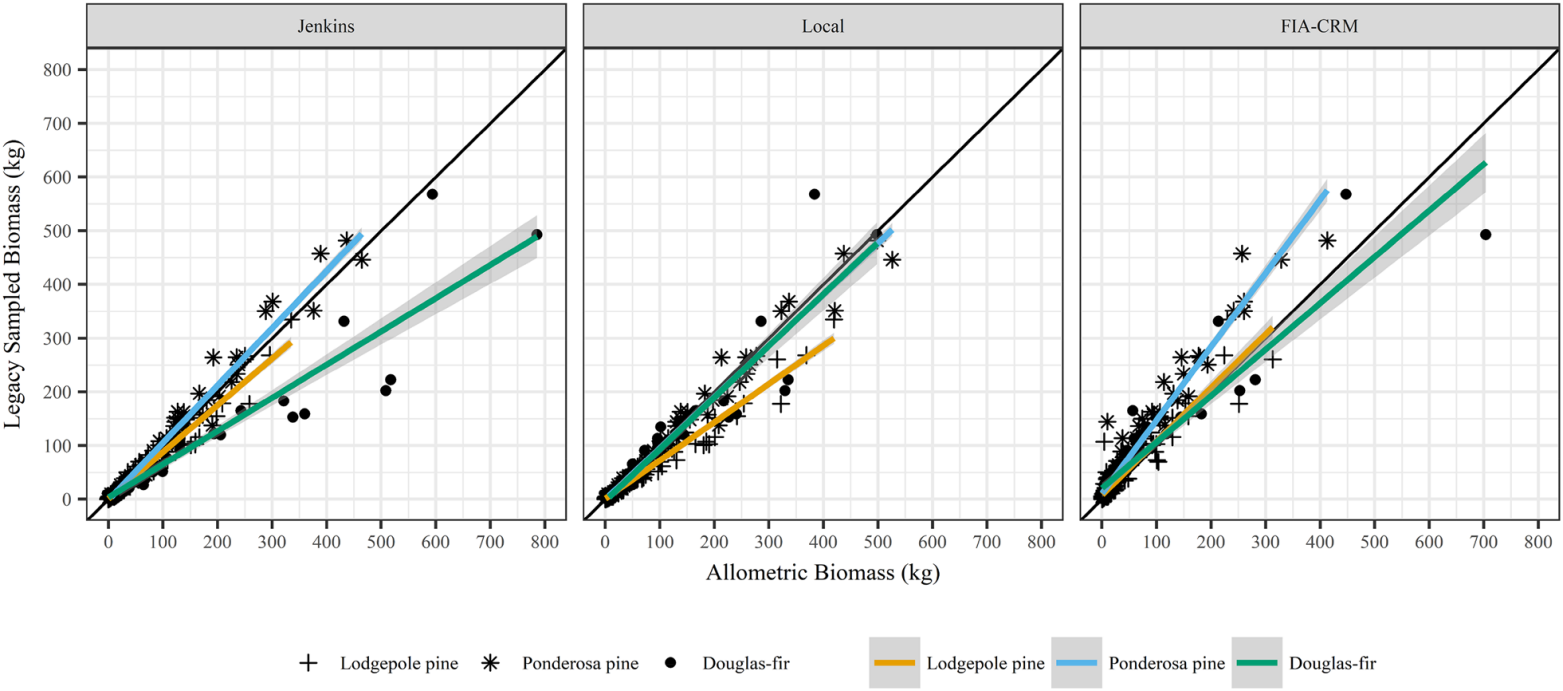
Scatter plots with regression lines and 95% confidence intervals comparing destructively-sampled biomass estimates from the legacy database to the three allometric equations used in this study (Jenkins et al. [11], local, and FIA-CRM). Comparisons are made for three species, as shown in the legend. The 1:1 line of exact agreement between Legacy sampled biomass and allometric biomass is shown by the black line for reference. All biomass estimates shown in the figure exclude foliage and stump biomass to align with the Legacy measurements used

We used data from 285 Legacy Tree Data trees to independently test the biomass predictions of the allometric equations developed in this study (“local”), Jenkins et al. [11], and the FIA-CRM (Table 1). The Legacy trees were smaller in terms of DBH and height than the trees that we destructively sampled and there were more low than high biomass Legacy trees (Fig. 3; Additional file 1: Table S6). Local equations had the lowest error and bias across all three species, although the bias was similar between local and Jenkins et al. [11] equations (Table 4). In general, error and bias for each set of allometric equations differed dramatically for each species. Error values were as high as 113.7% of the mean (Jenkins et al. [11] equations for Douglas-fir) and as low as 23.1% (local equations for ponderosa pine), while predictions were as biased as 54.0% (Jenkins et al. [11] equations for Douglas-fir) and had as little bias as − 4.5% (Jenkins et al. [11] equations for ponderosa pine; Table 4). The Jenkins et al. [11] equations predicted lodgepole pine biomass most accurately and performed similarly to the local equations for ponderosa pine. Local equations performed best for Douglas-fir. The FIA-CRM equations underpredicted biomass for all species, while local and Jenkins et al. [11] equations were less biased, but tended to overpredict biomass (Table 4; Fig. 3).

**Table 4:** Comparison of biomass estimates measured in the Legacy Tree Database and predictions by three allometric biomass equations for the Legacy trees: local allometrics presented in this study, Jenkins et al. [11], and the Forest Inventory and Analysis Component Ratio Method (FIA-CRM)

	All Species (n = 285)				Lodgepole pine (n = 114)				Ponderosa pine (n = 108)				Douglas-fir (n = 63)			
	RMSE		Bias		RMSE		Bias		RMSE		Bias		RMSE		Bias	
	kg	%	kg	%	kg	%	kg	%	kg	%	kg	%	kg	%	kg	%
Local	27.9	46.3	9.1	15.1	30.7	71.3	16.0	37.1	16.6	23.1	3.8	5.2	36.6	51.7	5.8	8.2
Jenkins	40.6	67.4	9.3	15.4	16.1	37.5	5.2	12.0	17.3	23.9	− 3.3	− 4.5	80.5	113.7	38.2	54.0
FIA-CRM	37.3	62.0	− 16.1	− 26.8	23.0	53.5	− 7.9	− 18.3	45.4	62.9	− 27.1	− 37.6	42.5	60.0	− 12.3	− 17.4

Negative bias values indicate that the allometric equations are under-predicting biomass compared to the Legacy Database biomass

**Fig. 4:**
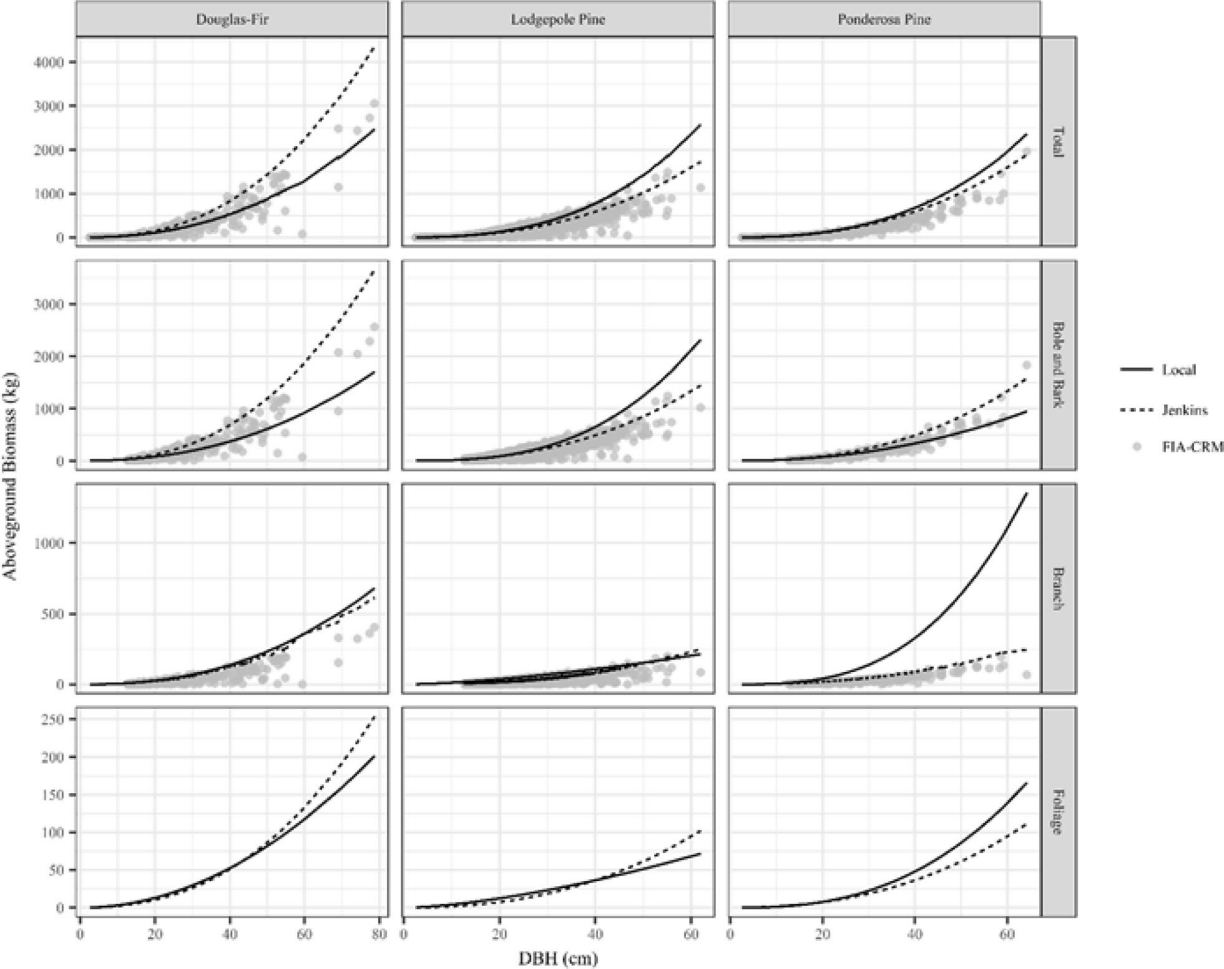
Comparison of the three sets of allometric equations used in this study (local equations presented in this study, Jenkins et al. [11], and Forest Inventory and Analysis Component Ratio Method [FIA-CRM]). Comparisons are made for the three species that were destructively sampled in this study (Douglas-fir, lodgepole pine, and ponderosa pine), the total tree biomass and three components. The figure shows biomass for the trees in the FIA plots used in this study. Total biomass includes stump biomass, but excludes foliage since FIA-CRM does not estimate foliage biomass. Bole and bark biomass are combined for the sake of direct comparison since FIA-CRM does not separate these components. This component represents bole and bark of the merchantable portion of the tree between the 1-foot stump and 4-inch top

Differences between allometric equations at the tree scale varied by species, component, and diameter (Fig. 4). Disagreement between allometric equations was minor for some species and components (e.g., total ponderosa pine biomass predicted by local and Jenkins) and large for others (e.g., total ponderosa pine biomass predicted by local and FIA-CRM; Fig. 4 and Additional file 3: Table S1). FIA-CRM biomass estimates have a range for a given tree diameter (the spread of gray points in Fig. 4) as opposed to a single line of biomass estimates like Jenkins and local equations because FIA-CRM biomass estimates are based on more factors than just diameter, such as height and tree breakage or rot. Jenkins allometric equations tend to predict the highest biomass values for Douglas-fir, while local equations predict the highest biomass for ponderosa and lodgepole pine. However, there is variability in this order by components. For example, local equations predict dramatically more ponderosa pine branch biomass than the other allometric equations, but Jenkins predicts the highest ponderosa pine bole and bark biomass. The absolute difference between allometric equations tended to increase with diameter and the relative difference had mixed trends (Fig. 4 and Additional file 3: Table S1). The relative difference sometimes increased with diameter but for other species and components, the relative difference decreased with diameter or remained relatively steady.

Engelmann spruce, subalpine fir, and aspen were all common in our plots, but were not destructively sampled in this study. Other allometric biomass equations were used for these species in the local biomass estimates for scaling to the plot and landscape-level. Engelmann spruce biomass from Landis and Mogren [52] and aspen biomass from Johnston and Bartos [53] both predicted higher biomass than FIA-CRM, but lower biomass than Jenkins et al. [11] (Table 5). The largest tree-level mean biomass difference for the non-focal species of this study was for Engelmann spruce between Jenkins et al. [11] and FIA-CRM (83.4 kg) and the largest mean relative difference was for subalpine fir (− 116.3%) between local (same as FIA-CRM for subalpine fir tree-scale comparisons) and Jenkins et al. [11].

**Table 5:** Comparison of aboveground tree biomass (stump included, foliage excluded for all allometric equations) for tree species common in our study area, but not destructively sampled

Species	Local—Jenkins		Local—FIA-CRM		Jenkins—FIA-CRM	
	Mean diff (kg)	Mean relative diff (%)	Mean diff (kg)	Mean relative diff (%)	Mean diff (kg)	Mean relative diff (%)
Engelmann spruce	− 20.2	− 15.6	67.8	33.4	83.4	41.2
Subalpine fir	− 60.8	− 116.3	NA	NA	60.8	44.2
Quaking aspen	− 35.1	− 30.0	12.4	23.3	47.9	41.2

Local biomass estimates for Engelmann spruce, subalpine fir, and aspen were made using allometric equations from Landis and Mogren [52], FIA-CRM, and Johnston and Bartos [53], respectively

The relative differences were calculated by dividing each tree biomass difference by the minuend. FIA-CRM was used to estimate tree-level biomass in both the local and FIA-CRM scenario, so their difference is not applicable

### Plot-Scale Biomass Variability

**Fig. 5:**
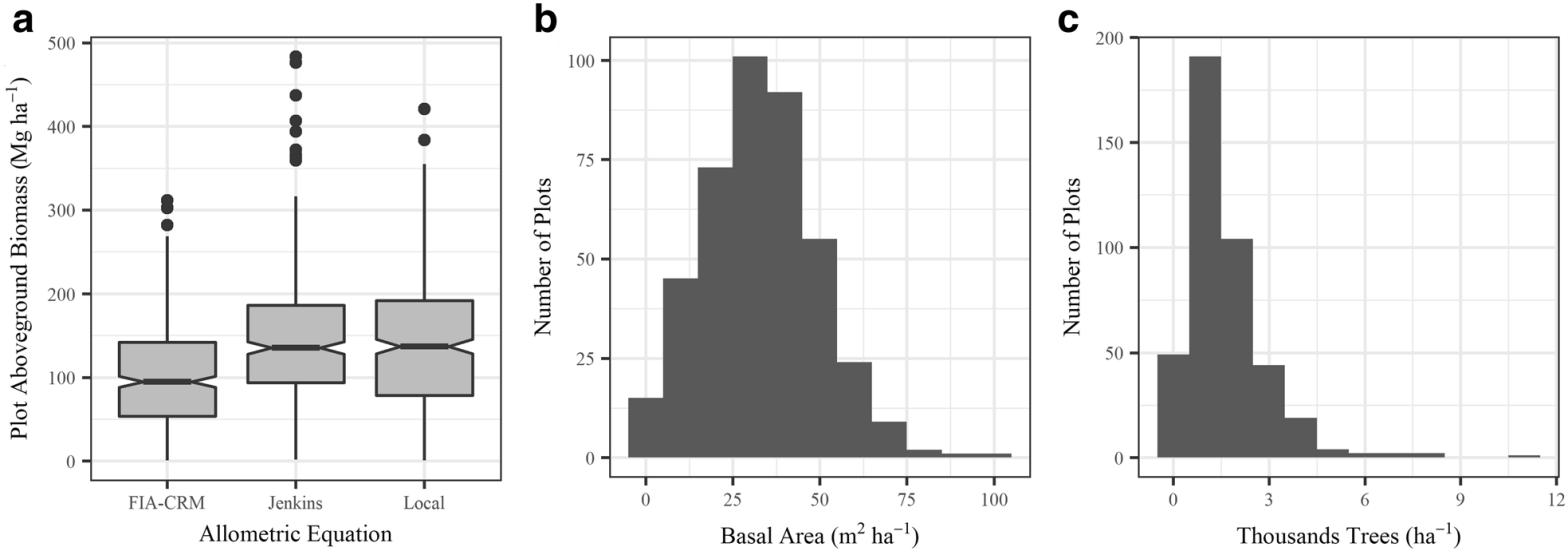
**a** Summary of aboveground tree biomass at Forest Inventory and Analysis (FIA) plots as estimated by three allometric equations. FIA-CRM = FIA Component Ratio Method (does not estimate foliage mass); Jenkins = Jenkins et al. [11]; local = equations presented in this study. **b** and **c**) Histograms of basal area and the number of trees per hectare at the FIA plots

We calculated biomass at 418 FIA plots with each set of allometric equations. Plots ranged from dense (max basal area = 95.2 m^2^ ha^−1^, max trees ha^−1^ = 11,411) to sparse (min basal area = 0.5 m^2^ ha^−1^, min trees ha^−1^ = 30), with an average basal area of 33.0 m^2^ ha^−1^ and 1608 trees per hectare (Fig. 5). Lodgepole pine accounted for 40.5% of the basal area, making it the most abundant species in these plots. Engelmann spruce (20.6%) and subalpine fir (17.1%) were also common, while aspen (7.6%), Douglas-fir (4.3%), ponderosa pine (3.5%), limber pine (2.0%), Utah juniper (1.4%), and pinyon pine (1.0%) made up small percentages of the plot basal area. Of the three species that we destructively sampled, ponderosa pine in the FIA plots tended to be the largest (mean average diameter = 23.2 cm, max = 64.3 cm, min = 2.5 cm), Douglas-fir in the middle (mean average diameter = 21.5 cm, max = 78.7 cm, min = 2.5 cm), and lodgepole pine the smallest (mean average diameter = 19.9 cm, max = 62.0 cm, min = 2.5 cm).

Biomass at the FIA plots tended to be highest when using Jenkins et al. [11] equations (mean biomass = 144.4 Mg ha^−1^), followed by local equations (mean biomass = 137.5 Mg ha^−1^) and then FIA-CRM (mean biomass = 100.2 Mg ha^−1^, Fig. 5). The differences between plot estimates made using Jenkins et al. [11] and FIA-CRM were the largest, followed by local equations and FIA-CRM differences (Table 6). Local equations and Jenkins et al. [11] plot biomass estimates were most similar, however differences between these two were larger when presented in terms of the absolute value of the biomass differences (Table 6). This reflects that plot biomass estimates made by local equations were sometimes higher and sometimes lower than Jenkins et al. [11] estimates. Differences between local and FIA-CRM and Jenkins et al. [11] and FIA-CRM changed little when the absolute values of the differences were considered, reflecting the consistent under-estimation of biomass by FIA-CRM. Random forests models of these differences in plot biomass estimates as a function of forest structure attributes showed that differences between allometric equations were larger in stands with higher basal area (Additional file 3: Figs. S1 to S4). Forest structure attributes explained between 56.7 and 86.1% of the variance in plot biomass differences (Additional file 3: Table S2). When the relative biomass between allometric equations was modeled, less of the variance was explained by stand structure and composition (39.0–55.3%).

**Table 6:** Summary of differences in plot biomass when calculated using different allometric equations: local (presented in this study), Forest Inventory and Analysis Component Ratio Method (FIA-CRM), and Jenkins et al. [11]

Allometric equations compared	Mean difference (Mg ha^−1^)	Mean of absolute differences (Mg ha^−1^)	Mean relative difference (%)
Local—Jenkins	− 6.9	25.9	− 13.0
Local—FIA-CRM	37.3	37.9	28.7
Jenkins—FIA-CRM	44.2	44.8	32.9

The mean of both the differences and the absolute value of the differences are presented as well as the mean relative difference

**Fig. 6:**
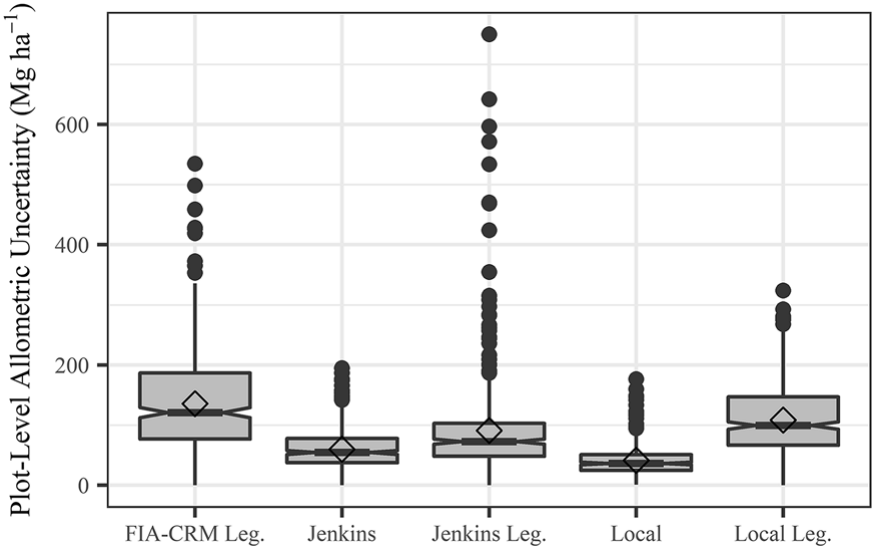
Boxplots showing plot-level allometric uncertainty for local equations (those presented in this study), Jenkins et al. [11], and the Forest Inventory and Analysis Component Ratio Method (FIA-CRM). Allometric error was evaluated in two ways for Jenkins et al. [11] and local equations: equation-derived evaluation and independent evaluation against Legacy Tree Data (“Jenkins Leg.” and “Local Leg.”). The FIA-CRM model was only evaluated against Legacy tree data (“FIA-CRM Leg.”). Notches on the boxplots show roughly a 95% confidence interval for the medians. Diamonds are the mean

Plot-scale allometric uncertainty was lower when utilizing equation-derived allometric errors, and was higher when propagating the independent Legacy evaluation (Fig. 6). The equation-derived local allometric equation error values resulted in the lowest plot-level uncertainty (mean plot RMSE = 41.5 Mg ha^−1^). However, when evaluated against the independent Legacy Tree data, Jenkins et al. [11] was lowest (mean plot RMSE = 90.9 Mg ha^−1^), followed by local equations (mean plot RMSE = 108.4 Mg ha^−1^) and FIA-CRM (mean plot RMSE = 135.7 Mg ha^−1^).

### Landscape-scale biomass variability

**Fig. 7:**
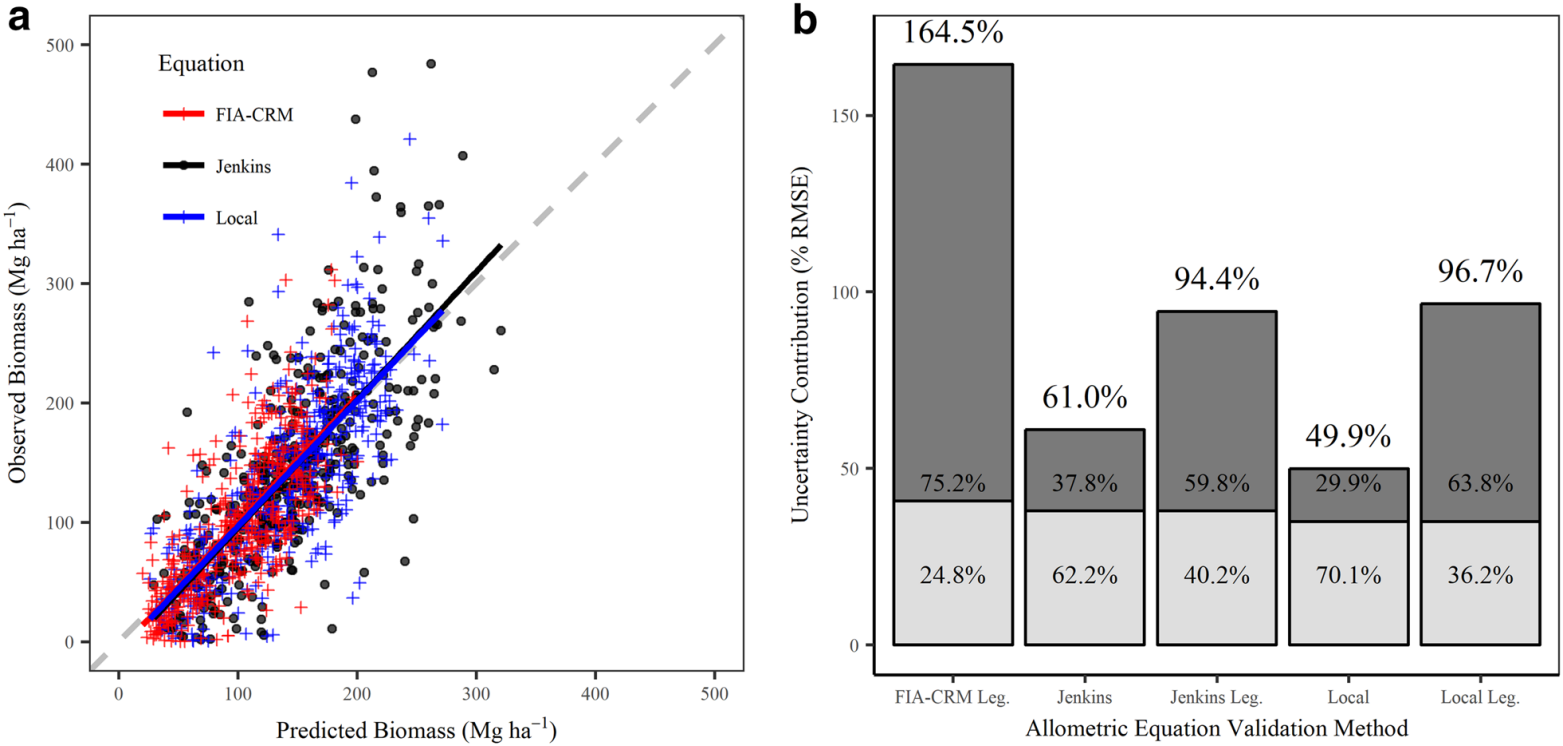
**a** Observed plot aboveground biomass values and those predicted by the random forest models for generating biomass maps in three allometric biomass equation scenarios: local equations (those presented in this study), Jenkins et al. [11], and the Forest Inventory and Analysis Component Ratio Method (FIA-CRM). Dashed grey line is the 1:1 line representing perfect model fit.** b** Uncertainty contributions from the allometric model (dark grey) and random forest prediction (light grey). The printed percentages within each bar are the allometric and prediction uncertainties relative to total uncertainty. Total uncertainty is printed at the top of each bar. Allometric error was evaluated in two ways for Jenkins et al. [11] and local equations: equation-derived and independent evaluation against Legacy tree data (“Jenkins Leg.” and “Local Leg.”). The FIA-CRM model was only evaluated against Legacy tree data (“FIA-CRM Leg.”) because we were unable to locate FIA-CRM equation-derived errors

We developed three maps of standing aboveground tree biomass in 2001 using random forests models: one map for each set of allometric biomass equations. The three models performed similarly with 53.77- 59.36 percent variation explained and RMSEs ranging from 40.8 to 54.8 Mg ha^−1^ and low bias (Table 7). The Normalized Difference Infrared Index (NDII) was an important variable in all models, and the Landsat 7 blue band (band 1) and digital elevation model were also important predictor variables (Table 7). Consistent with other studies that map biomass using passive remote sensing, the biomass predictions saturate in all three maps (Fig. 7a). Models built from allometric equations that predict higher biomass in the FIA plots (local and Jenkins et al. [11]) saturate at slightly higher levels than FIA-CRM, which estimated lower biomass at the FIA plots.

**Table 7:** Out-of-bag model evaluation metrics (pseudo R^2^ and RMSE) from the Random Forest aboveground biomass models

Allometric Equation	RMSE	RMSE percent of mean	Pseudo R^2^	Percent Bias	Number of Predictors (Top 3 Predictors)	mtry	ntrees
Local	48.1	35.0	0.5936	0.9	11 (Band 1, NDII, DEM)	8	2000
Jenkins	54.8	37.9	0.5377	0.9	10 (NDII, DEM, Band 1)	5	1500
FIA-CRM	40.8	40.8	0.5463	1.1	9 (NDII, Band 2 texture_5 × 5 mean, PAS)	4	1000

These values are the prediction errors only, and do not include allometric error. *FIA-CRM* Forest Inventory and Analysis Component Ratio Method, *RMSE* root mean square error, *NDII* normalized difference infrared index, *DEM* digital elevation model, *PAS* precipitation as snow, *mtry* number of predictor variables randomly sampled at each split in model, *ntrees* number of trees grown in model

We evaluated the contribution of two main sources of uncertainty to the biomass predictions: allometric error and prediction error from mapping biomass (Fig. 7b). Total uncertainty was lowest for models constructed using equation-derived evaluation of local allometric equations (49.9%) and was highest for independently evaluated FIA-CRM models (164.5%; Fig. 7b). Model prediction error was relatively consistent across all models. The variability in total uncertainty was driven by allometric uncertainty, which ranged from 29.9 to 75.2% of the total uncertainty (Fig. 7b). Allometric errors were less than prediction errors only when equation-derived allometric errors were used.

**Fig. 8:**
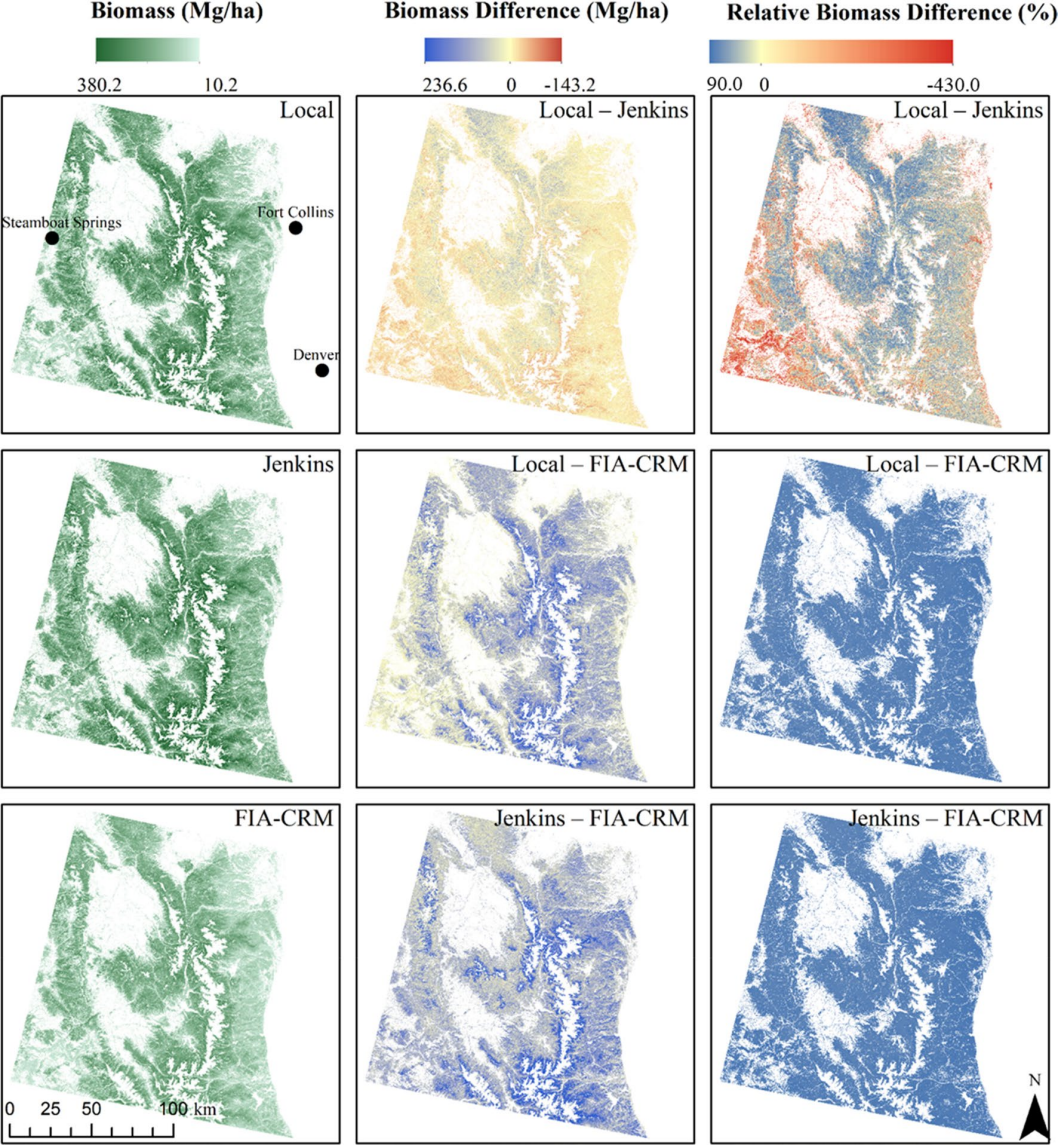
Left column: Biomass maps created using three sets of allometric biomass equations: local equations presented in this study, Jenkins et al. [11], and the Forest Inventory and Analysis Component Ratio Method (FIA-CRM). Middle and right columns: Maps of the difference and relative difference between the biomass maps, respectively. Note that FIA-CRM biomass estimates do not include foliage mass, but local and Jenkins et al. [11] maps do

**Fig. 9:**
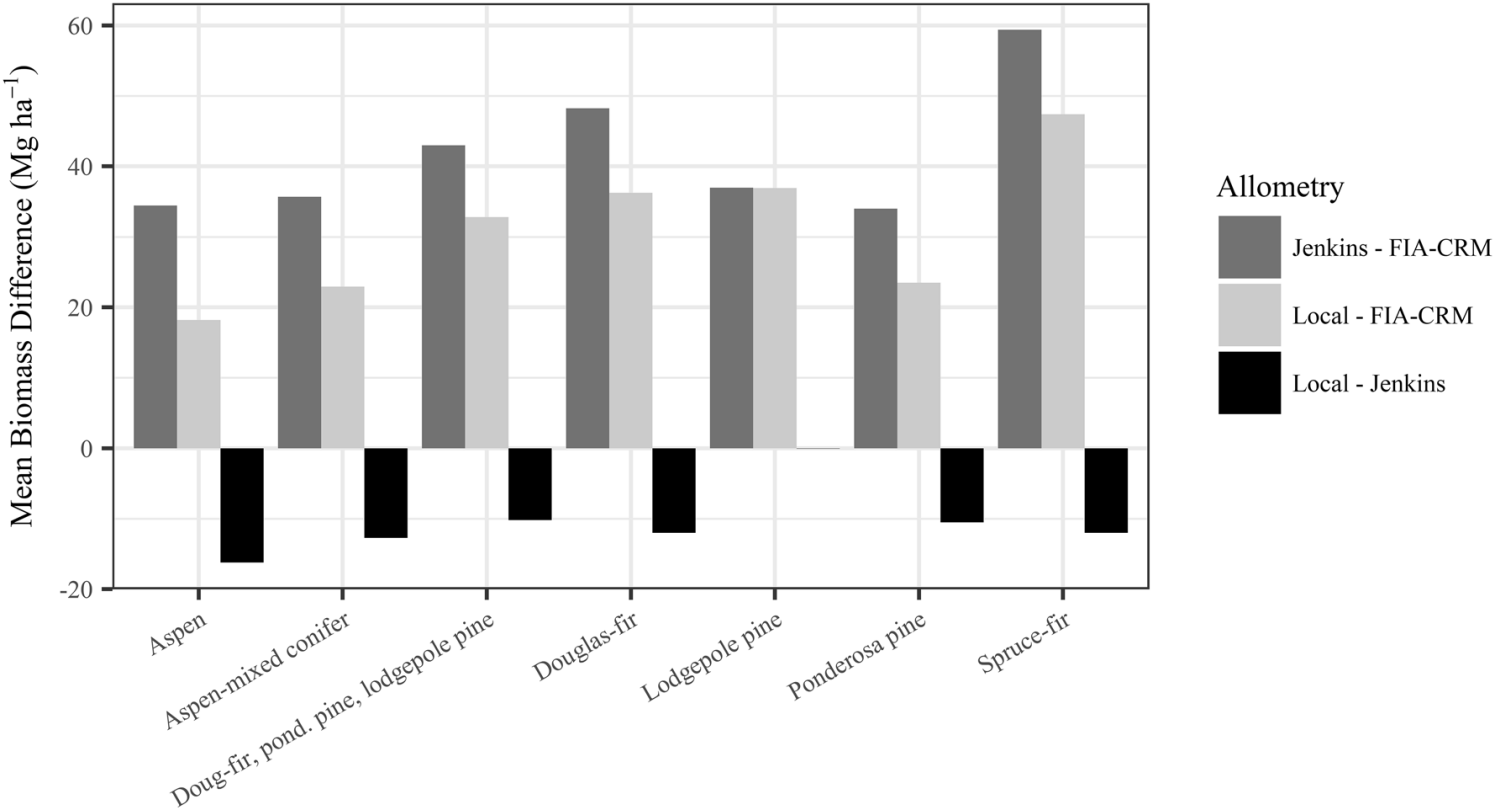
Mean difference in biomass between maps produced from three sets of allometric biomass equations for the most common forest types in our study area. The mean was taken of all pixels within each forest type. Forest types are derived from LANDFIRE Existing Vegetation Type version 1.0.5—forest type names are simplified for this figure. Note that mapped Forest Inventory and Analysis Component Ratio Method (FIA-CRM) biomass estimates do not include foliage, but local and Jenkins et al. [11] estimates do include foliage

Across all forested areas in our study area, maps generated using local allometric biomass equations estimate 2.066 billion Mg of standing aboveground biomass, while maps based on Jenkins et al. [11] and FIA-CRM equations estimate 2.224 billion Mg and 1.502 billion Mg, respectively. The maps based on Jenkins et al. [11] showed 7.6% more biomass than the local maps, while FIA-CRM maps showed 27.3% less biomass than local maps. These three biomass maps were differenced to highlight areas of agreement and disagreement in the predicted amount of aboveground forest biomass (Fig. 8). Differences between biomass in a given pixel were as large as 236.6 Mg ha^−1^. The largest differences between “Jenkins – FIA-CRM” and “local – FIA-CRM” were in spruce-fir forests (Fig. 9). Local and Jenkins et al. [11] predictions were remarkably similar for lodgepole pine forests and were most different for aspen forests (Fig. 9).

## Discussion

Allometric equation selection is critical for accurately estimating regional biomass stocks. We evaluated the variability and accuracy of aboveground forest biomass estimates from three sets of allometric equations at the tree, plot, and landscape scale. Destructive sampling of even as few as ten trees per species generated relatively reliable allometric equations for our study area compared to existing equations. In an independent evaluation of these local equations, Jenkins et al. [11], and FIA-CRM, we found the local equations performed best for Douglas-fir and comparably to Jenkins et al. [11] for ponderosa pine. Our local lodgepole pine equations had the highest error and bias of the allometric equations tested. While it is reasonable that the local equations would perform strongly in the areas near our destructive sampling sites, we were also surprised given our low sample sizes and potential for high bias [71]. Due to the low sample size sampled across small areas, the allometric equations presented in this study should be used cautiously if applied in other regions. More accurate local allometrics could be developed by sampling additional trees representing a variety of genetic, abiotic, and biotic conditions and by incorporating more predictor variables in the allometric equations such as height and crown ratio.

Accuracy of each of the equations differed substantially between species. For example, Jenkins et al. [11] performed well compared to the Legacy Tree Data for lodgepole pine (RMSE = 16.1 kg; Table 4), but poorly for Douglas-fir (RMSE = 80.5 kg). Allometric performance should be tested for each species of interest when possible. The variability in biomass estimates across diameters (i.e., higher differences between allometrics at some diameters) suggests that equations should also be tested across the diameter range being used.

The FIA-CRM equations, which are commonly used for forest carbon accounting [e.g., 15], consistently under-estimated biomass and generated the lowest estimates at the tree, plot, and landscape scales. Other studies have also found FIA-CRM to under-estimate biomass [e.g., 13, 72]. The FIA-CRM plot and landscape-level estimates did not include foliage biomass while estimates from the other allometrics did include foliage. Foliage accounted for an average of 14% of total aboveground biomass for the trees we destructively sampled. Differences between equations at the plot scale (28.7% mean difference between local and FIA-CRM and 32.9% difference between Jenkins and FIA-CRM) and across the entire study area (27.3% difference between local and FIA-CRM and 32.5% difference between Jenkins and FIA-CRM) are influenced by the exclusion of foliage in FIA-CRM estimates. However, biomass differences exceeded what can be attributed to the exclusion of foliage, indicating that FIA-CRM underestimates the biomass of other components. Foliage was excluded for the comparison of all allometric predictions to Legacy Trees to reduce impacts of the lack of foliage on FIA-CRM uncertainty propagation.

Biomass differences between equations varied widely across species, DBH, and component, indicating that total tree biomass errors can’t be assumed to represent errors for a single component, or errors for another species or size class. For example, branch biomass predictions varied between equations (75% difference between local and Jenkins et al. [11]) much more than total biomass (14% difference) for large ponderosa pine (40–60 cm DBH; Fig. 4; Additional file 3: Table S1). This also highlights the potential pitfalls and strengths of local allometric equations. The local equations predict high branch biomass compared to other equations, potentially reflecting true differences in growth form between our study area and the areas from which trees were sampled to develop these other equations. However, the high branch estimates could also be the result of sampling bias. We sampled ponderosa pine in a variety of stand densities, but several of our large trees were more open grown and thus had more branch biomass, contributing to the high branch biomass predictions.

The differences between allometric equation biomass predictions were frequently, but not always, largest for the biggest (60–80 cm DBH) trees (Additional file 3: Table S1). This reflects an issue common for biomass allometry: large trees have the most biomass and greatest variation in growth form, but are rarely measured because they are the most difficult and expensive to sample [73]. We had only one destructively sampled ponderosa pine tree in this upper diameter range, and lacked any trees this size for lodgepole pine or Douglas-fir. While it is problematic to predict outside the diameter range of sampled trees, this practice is commonplace in biomass assessments because few alternatives exist for most species and locations. Improved allometric equation accuracy for large trees is needed to improve forest biomass estimates [39]. The inclusion of large trees in the Jenkins et al. [11] equations likely make the large tree biomass predictions more reliable than the local equations. However, allometric equations that don’t utilize tree height can overpredict large diameter tree biomass [13].

Plot-level biomass estimates diverged with increasing basal area. Engelmann spruce-subalpine fir forests are some of the higher basal area forests in our study area. The maps (Fig. 8) and summaries of biomass differences by forest type (Fig. 9) both show high disagreement in these spruce-fir forests between all equations, highlighting it as a forest type where allometric equation selection is particularly important. Independent evaluation of the allometric equations for these species is needed to determine which allometric equation is best suited for this forest type. Allometric choice is also important in the lower elevation montane forests due to the high relative biomass difference between Jenkins and local equations (Fig. 8). The use of our local equations is advised for these montane forests in our study area based on the favorable performance of the local equations relative to the Legacy Tree data for Douglas-fir and ponderosa pine.

The tree biomass measurements needed to independently evaluate allometric accuracy are rare and valuable. These data are typically unavailable for a particular area or species, or are used in the development of the allometric equations themselves. Resources such as the Legacy Tree Database [40] and efficient non-destructive biomass sampling methods [44, 74] make independent allometric validation more feasible. Independent tree biomass datasets come with their own difficulties and biases due to the potential for biased sampling and inconsistent destructive sampling methodologies and component definitions. For example, we converted green mass to dry mass and adjusted which components were included in our independent evaluation dataset. We also had a disproportionately high number of small trees in the Legacy Data (Additional file 1: Table S6) that likely had the effect of underestimating allometric uncertainty of all allometric equations since allometric error tends to be less for smaller trees. We encourage biomass studies to openly share data (Additional file 4) to enable improved evaluation of existing allometric biomass equations and for updating or building new equations. Even if independent data are not available for all species within a study area, the most common species can be prioritized as they have a larger influence on biomass uncertainty than less abundant species.

Independently evaluating tree-level allometry increased uncertainty estimates. Errors reported with allometric equations reflect allometric performance relative to samples used to build the equations, not necessarily for the application area. These samples may be a small, localized dataset (local equations) or from a large geographic area containing multiple species [11]. When using equation-derived errors, local allometric equations had 11% lower total uncertainty than the Jenkins et al. [11] equations (Fig. 7b). However, evaluating both allometric equations with independent data resulted in similar and higher overall uncertainties. The increase in plot and landscape level uncertainty resulting from independent evaluation is likely an underestimate since we only had data to independently evaluate three species. To our knowledge, allometric errors are not reported for FIA-CRM equations, so independent evaluation is the only appropriate way to quantify FIA-CRM allometric error. Comparing allometric biomass predictions to independent biomass observations from the application area enables improved estimates of allometric uncertainty, and guides selection of the best allometric equations for a particular region or application.

Reporting remote sensing model prediction error alone, as is common in many studies, insufficiently represents biomass estimation uncertainty. Allometric error should be considered, and, if possible, should be based on independent evaluation of the allometric equations from the population of interest. The accuracy of each biomass map in this study appeared similar if only considering remote sensing model prediction error, but differed widely once allometric uncertainty was propagated (Fig. 7b). Ignoring allometric uncertainty and only reporting model prediction uncertainty would have represented as little as a quarter of the total uncertainty, reflecting a false confidence in the biomass maps. Just as FIA-CRM had the highest tree-level errors, biomass maps built using FIA-CRM had the highest total uncertainty. Propagation of allometric uncertainty from an independent dataset revealed very high biomass estimate uncertainties, and improved accuracy by informing the selection of the most accurate allometric equations.

## Conclusion

Allometric equation selection is a dominant influence on forest aboveground biomass estimates at the tree, plot, and landscape scale. Unless allometric uncertainty is propagated, total error in biomass estimates will be underestimated and uncertainty of estimates made with different allometric equations may look deceivingly similar, giving false confidence in mapped estimates of biomass. Allometric uncertainty can exceed the remote sensing model prediction uncertainty. Furthermore, regional evaluation is needed to quantify allometric performance in the study area. We found reported allometric equation error to underestimate error compared to an independent, regional, tree-level biomass validation dataset. Total uncertainty was comparable between estimates made using nationwide allometric equations [11] and local, low sample size equations. Both outperformed FIA-CRM equation uncertainty. Future efforts should incorporate other sources of uncertainty not considered here (e.g. diameter and height measurements). Remote sensing model prediction uncertainty will be reduced as algorithms improve and LiDAR and synthetic aperture radar become more widely available [e.g., 75], increasing the relative contribution of allometric uncertainty to total uncertainty [39]. Efforts to quantify and reduce allometric uncertainty are also needed. Data repositories of individual tree biomass data (such as the Legacy Tree Database used in this study) will be key in building more robust and independently evaluated allometric equations at regional-scales. Additional destructive sampling and refinement of nondestructive sampling methods will help quantify and reduce allometric uncertainty.

## Supplementary information

**Additional file 1.** Additional information for the methods; Tables and text detailing additional information about methods, such as destructive sampling, component calculation, branch wood and foliage component estimation, predictor variables used for biomass mapping, and a comparison of trees from the various data sources in this study.

**Additional file 2**. Results of destructive sampling and oven drying; Tables and figures presenting mass and moisture content of each component from destructive sampling of Douglas-fir, lodgepole pine, and ponderosa pine.

**Additional file 3.** Biomass variability between allometric equations; Tables and figures characterizing tree and plot-level biomass differences between the three allometric equations evaluated in this study.

**Additional file 4.** Destructive sampling data; Data from each tree destructively sampled for this study showing component biomass (kg), diameter at breast height (cm), and height (m) as well as basal area (m^2^ ha^−1^) and tree density (number of trees ha^−1^) of 7.32 m radius plots measured around each tree.

## Data Availability

The destructive sampling data supporting the conclusions of this article are included within the article and its additional files (see Additional file 4). The Forest Inventory and Analysis data supporting the conclusions of this article are available online at https://www.fia.fs.fed.us/(although exact plot locations are not publicly available). Landsat data supporting the conclusions of this article are available from https://earthexplorer.usgs.gov/. Legacy Tree Data supporting the conclusions of this article are available from http://www.legacytreedata.org/.

## Abbreviations

REDD+: Reducing Emissions from Deforestation and forest Degradation
DBH: Diameter at breast height
U.S.: United States
FIA-CRM: Forest Inventory and Analysis Component Ratio Method
EPA: Environmental Protection Agency
LiDAR: Light Detection and Ranging
FIA: Forest Inventory and Analysis
ETM+: Enhanced Thematic Mapper Plus
asl: Above sea level
RMSE: Root mean square error
L1T: Level 1 Terrain-corrected
CFMask: C Function of Mask
ntree: Number of trees
mtry: Number of parameters considered at each node
VSURF: Variable Selection Using Random Forest
NF: National Forest
CO: Colorado
PSME: Douglas fir (*Pseudotsuga menziesii)*
PICO: Lodgepole pine (*Pinus contorta*)
PIPO: Ponderosa pine (*Pinus ponderosa*)
NDII: Normalized Difference Infrared Index
DEM: Digital elevation model
PAS: Precipitation as snow
