# Supplementary material for: Variability and uncertainty in forest biomass estimates from the tree to landscape scale: the role of allometric equations
Source: Carbon Balance Manag. 2020 May 14;15:8. doi: 10.1186/s13021-020-00143-6 (PMC7227279; doi:10.1186/s13021-020-00143-6)
Supplement: Supplementary file 1 — Additional file 1. Additional information for the methods; Tables and text detailing additional information about methods, such as destructive sampling, component calculation, branch wood and foliage component estimation, predictor variables used for biomass mapping, and a comparison of trees from the various data sources in this study. [file 13021_2020_143_MOESM1_ESM.docx]

## Additional File 1: Additional information for the methods

## Tables and text detailing additional information about methods, such as destructive sampling, component calculation, branch wood and foliage component estimation, predictor variables used for biomass mapping, and a comparison of trees from the various data sources in this study.

Additional details about the methods

Destructive Sampling

We collected size and mass measurements in the field for the bole, bark, crown (defined as the portion of the tree between the first live branch and the 10.2 cm top), and top of the tree (everything above the 10.2 cm top) using digital scales (OHAUS Valor 1000 model V11P6 precision scale, maximum 6 kg, least count 0.001 kg; Tree LVS 700 large scale, maximum 320 kg, least count 0.1 kg). Bole, bark, branch wood, and branch foliage subsamples were collected and oven dried at 105°C [1] to determine moisture content and component biomass. Disks cut from the top of 1.2 m bole segments served as the bole and bark subsamples. We measured the weight and dimensions of the disk, with and without bark. A 10 cm strip of bark was measured, weighed, and kept for oven-drying. Six live branches—two randomly selected from the lower, middle, and upper third of the crown—were collected from each tree as wood and foliage subsamples. For small trees with fewer than six branches below the 10.2 cm top, we randomly selected four subsample branches from the 15 lowest branches. Only a portion of branch wood was kept for branches >5 kg to expedite oven drying, but all foliage was retained. Dead branches were also subsampled for moisture content (n = 10 lodgepole pine, n = 12 ponderosa pine, n = 14 Douglas-fir).

### Component Biomass Calculation

Bole and bark component biomass were calculated using the disks and 1.2 m bole segments. We calculated the bark mass per unit bole surface area from the disks, and then averaged across all disks for an individual tree. This average for each tree was multiplied by the surface area of each segment and summed to obtain bark wet mass. Bole wood wet mass was calculated by subtracting the bark wet mass from the field-measured four-foot segments. The percentage moisture content from the bark subsamples and disks were applied to obtain bark dry mass and bole wood dry mass, respectively. The disk moisture content was also used to calculate the dry mass of the top main stem.

We developed regression equations for each species to estimate branch foliage and wood mass for each crown branch [1–4]. The first step was to predict the length for the two thirds of the crown branches where length was not measured so that length could be considered as a predictor variable when estimating branch foliage and wood mass. We generated a multiple regression equation for each species to predict branch length considering the following predictor variables in linear and log-linear equations: branch diameter, tree diameter at breast height, stem height at base of branch, tree height, relative branch depth [5], crown width at widest axis, and height to first branch. Equations expected to perform well were developed a priori (Table 1, Additional File 1) and were then modified based on model evaluation statistics and reduction of predictor variables correlated by 0.70 or more [6]. Equations were selected that performed best based on adjusted R^2^ and root mean square error (RMSE) and that met assumptions of residual normality and homoscedasticity. The same method used to select the branch length equations was used to determine the best equation for branch foliage and branch wood wet mass. We considered the same predictor variables with the addition of branch length. The top models (Table 2, Additional File 1) were used to estimate branch foliage and branch wood wet mass for each branch of each destructively sampled tree. To account for potential downward bias when back-transforming the log transformed model predictions, a correction factor can be applied [7]. However, this correction factor can also introduce bias, particularly with low sample size equations. So, as suggested by other studies [8], we reported but did not apply the correction factor (Table 3, Additional File 1).

These estimates of branch wood and foliage mass for each crown branch served as the basis for calculating the branch and foliage component of the whole tree. We generated a ratio of wood to crown wet mass and foliage to crown wet mass for each tree using the sum of branch foliage and wood from the branch-by-branch estimation. These ratios were applied to crown and top branch mass, then the water weights were subtracted using a tree-specific water content for each component. For trees too small to have crown branches, we calculated branch and foliage ratios from the subsampled branches. This approach of using the wood and foliage ratios ensured that the sum of wood and foliage biomass equaled the total crown and top mass measured in the field. Dry top main stem mass and dry dead branch mass were then added to the branch wood component.

Allometric Equation Calculation

While including height as a predictor variable with DBH can increase the accuracy of allometric equations, we use DBH as the only predictor variable in these allometric equations for several reasons. Diameter at breast height is more commonly and accurately measured than tree height, so using DBH only makes the equations more widely applicable and less prone to measurement error [9]. Additionally, the small sample size of destructively sampled trees did not capture the variation in tree height for trees with similar diameters.

Details about FIA-CRM Component Biomass Calculation

Bark volume is estimated as a percentage of bole volume and is converted to bark biomass using specific gravity. Biomass of the entire tree, merchantable bole, and belowground biomass is calculated using equations from Jenkins et al. [8]. Then, equations from Raile [10] are used to calculate stump volume. Top biomass is calculated as the difference between total aboveground biomass and all other components. Lastly, an adjustment factor calculated as the ratio of bole biomass between the regional volume based-estimate and the Jenkins et al. [8] estimate is applied to adjust the biomass of all tree components. The FIA-CRM equations do not estimate foliage biomass. Sapling (trees < 12.7 cm [5.0 inches] DBH) biomass is calculated as the product of total aboveground biomass, excluding foliage, from Jenkins et al. [8] and a sapling adjustment factor found in Heath et al. [11]. This same method is used for estimating aboveground biomass of woodland species (i.e., trees where diameter is measured at root collar) that are less than 12.7 cm (5.0 inches). For woodland trees greater than or equal to 12.7 cm (5 inches), biomass is calculated from volume just as it is for tree boles, and component biomass is not calculated.

Predictor Variables for Biomass Mapping

Texture statistics were generated from grey-level co-occurrence matrices using the glcm package in R [12]. We made the following texture metrics for ETM+ bands 1, 2, 3, 4, 5, and 7 on a 3x3, 5x5, 7x7, 9x9, and 11x11 window and a (1,1) shift: mean, variance, homogeneity, contrast, dissimilarity, entropy, second moment, and correlation. Some of these texture measures have shown promise in other studies relating Landsat imagery to forest biomass [13–16]. We generated each combination of band, window size, and texture measures because the relationship between texture and forest biomass varies with many factors including vegetation type, imagery, and window size.

We generated additional topographic, geomorphometric, and climatic predictor variables that have been shown to correlate with tree species and biomass distributions [17,18]. Elevation, slope, northness, and eastness were derived from the National Elevation Dataset [19–21]. Using the Spatial Analyst [22] and Geomorphometry and Gradient Metrics Toolboxes [23], we also generated the compound topographic index, heat load index, curvature, and slope position and roughness on a 3x3, 6x6, 9x9, 12x12, 15x15, 21x21, and 27x27 window size. Climate normals (1961 – 1990) were downscaled to a 30 m x 30 m spatial resolution to match the scale of other rasters [24].

Variable Selection Using Random Forest (VSURF)

Variable Selection Using Random Forest (VSURF) was used to select our variables for modeling biomass across the landscape [25]. VSURF first ranks all variables by the variability in their variable importance score across 50 random forest runs. More important variables have higher variability than less important variables. Variables falling below an importance threshold are removed. An “interpretation” set of variables is next determined from this reduced set of variables by running 25 random forest runs with the top variable and then adding the next-most-important variable one-by-one and rerunning the models. The interpretation variables are those from the simplest model of the models with an out-of-bag error within one standard deviation of the minimum error. A “prediction” set of variables is selected by adding interpretation variables step-wise, and only keeping variables that reduce the out-of-bag error significantly more than adding a noisy variable. The VSURF procedure resulted in an interpretation set of variables each with a relationship to aboveground biomass and a prediction set with reduced redundancy suitable for prediction.

Modeling Plot-Scale Biomass Differences

At the plot scale, we identified the stand characteristics most correlated with biomass estimate differences between different sets of allometric equations. This was done using a random forest model to predict the plot biomass difference between allometric equations using stand structure and composition predictor variables. The following predictors were considered: number of trees per hectare, basal area, average tree diameter at breast height, and basal area by species of the most common species in the plot data (lodgepole pine, Engelmann spruce, subalpine fir, aspen, Douglas-fir, and ponderosa pine). We also considered the proportion of total basal area for these same species, but removed them from the model due to high correlation with the species absolute basal area values and the sensitivity of variable importance metrics to correlated predictor variables. We calculated variable importance and generated partial dependence plots [26] to characterize relationships between stand structure and allometric biomass equation differences.

Calculating Dry Mass of Sánchez Meador [27] Trees

Only green mass was reported for 212 of the 285 Legacy trees used in our study [27]. For these trees, we converted above-stump green mass to dry mass using moisture contents and component proportions from our destructive sampling. A species-specific, whole-tree water percentage was calculated as the mean of each component’s moisture content (Table 1, Additional File 2) weighted by the percentage of tree biomass in each component (Fig. 1, Additional File 2). Foliage mass was included but was not reported as a separate component for these trees sampled by Sánchez Meador [27], so we calculated foliage using our local equations and subtracted it from the above-stump dry weight to obtain a value comparable to the dry wood and bark mass used for trees from Reid et al. [28] and Tossey [29].

Table S1: A priori equations tested for predicting branch length, foliage, and branch wood for individual branches of each species. Several additional species-specific equations were also tested (not shown).

| Branch Length Equations (m) | Branch Wood and Foliage Equations (g) |
| --- | --- |
| β_0_ + β_1_B_ij_ + β_2_C_i_ | β_0_ + β_1_B_ij_ + β_2_L_ij_ |
| β_0_ + β_1_B_ij_ + β_2_C_i_ + β_3_R_ij_ | β_0_ + β_1_B_ij_ + β_2_L_ij_ + β_3_D_i_ |
| β_0_ + β_1_B_ij_ + β_2_R_ij_ + β_3_G_i_ + β_4_D_i_ | β_0_ + β_1_B_ij_ + β_2_L_ij_ + β_3_R_ij_ |
| β_0_ + β_1_B_ij_ + β_2_H_i_ + β_3_S_ij_ | β_0_ + β_1_B_ij_ + β_2_C_i_ + β_3_S_ij_ |
| β_0_ + β_1_B_ij_ + β_2_D_i_ + β_3_C_i_ + β_4_S_ij_ | β_0_ + β_1_B_ij_ + β_2_D_i_ + β_3_R_ij_ + β_4_G_i_ |
| β_0_ + β_1_B_ij_ + β_2_D_i_ + β_3_C_i_ | β_0_ + β_1_B_ij_ + β_2_L_ij_ + β_3_H_i_ |
| β_0_ + β_1_ ln(B_ij_) + β_2_ ln(C_i_) | β_0_ + β_1_B_ij_ + β_2_S_ij_ + β_3_C_i_ + β_4_D_i_ |
| β_0_ + β_1_ ln(B_ij_) + β_2_ ln(C_i_) + β_3_ ln(R_ij_) | β_0_ + β_1_ ln(B_ij_) + β_2_ ln(L_ij_) |
| β_0_ + β_1_ ln(B_ij_) + β_2_ ln(R_ij_) + β_3_ ln(G_i_) + β_4_ ln(D_i_) | β_0_ + β_1_ ln(B_ij_) + β_2_ ln(L_ij_) + β_3_ ln(D_i_) |
| β_0_ + β_1_ ln(B_ij_) + β_2_ ln(H_i_) + β_3_ ln(S_ij_) | β_0_ + β_1_ ln(B_ij_) + β_2_ ln(L_ij_) + β_3_ ln(R_ij_) |
| β_0_ + β_1_ ln(B_ij_) + β_2_ ln(D_i_) + β_3_ ln(C_i_) + β_4_ ln(S_ij_) | β_0_ + β_1_ ln(B_ij_) + β_2_ ln(C_i_) + β_3_ ln(S_ij_) |
| β_0_ + β_1_ ln(B_ij_) + β_2_ ln(D_i_) + β_3_ ln(C_i_) | β_0_ + β_1_ ln(B_ij_) + β_2_ ln(D_i_) + β_3_ ln(R_ij_) + β_4_ ln(G_i_) |
|  | β_0_ + β_1_ ln(B_ij_) + β_2_ ln(L_ij_) + β_3_ ln(H_i_) |
|  | β_0_ + β_1_ ln(B_ij_) + β_2_ ln(D_i_) + β_3_ ln(S_ij_) + β_4_ ln(C_i_) |

j^th^ branch on the i^th^ tree

L= branch length (m); D = tree diameter at breast height (cm); B = branch diameter (cm); C= width of crown at widest axis (m); S = height where branch meets main stem (m); R = relative branch depth; H= tree height (m); G = height to first branch (m)

Table S2: Model form selected for regression models used to predict branch length, foliage, and branch wood for individual branches.

| Species | Component | Formula |
| --- | --- | --- |
| Lodgepole Pine | Branch Length | L_ij_= β_0_ + β_1_B_ij_ + β_2_D_i_ + β_3_C_i_ |
|  | Foliage | ln(F_ij_)= β_0_ + β_1_ ln(B_ij_) + β_2_ ln(S_ij_) + β_3_ ln(R_ij_) |
|  | Wood | ln(W_ij_)= β_0_ + β_1_ ln(B_ij_) + β_2_ ln(L_ij_) + β_3_ ln(H_i_) |
| Ponderosa Pine | Branch Length | L_ij_= β_0_ + β_1_B_ij_ + β_2_C_i_ + β_3_S_ij_ + β_4_G_i_ |
|  | Foliage | ln(F_ij_)= β_0_ + β_1_ ln(B_ij_) + β_2_ ln(D_i_) + β_3_ ln(R_ij_) + β_4_ln(G_i_) |
|  | Wood | ln(W_ij_)= β_0_ + β_1_ ln(B_ij_) + β_2_ ln(S_ij_) + β_3_ ln(C_i_) |
| Douglas-Fir | Branch length | L_ij_= β_0_ + β_1_B_ij_ + β_2_D_i_ + β_3_G_i_ |
|  | Foliage | ln(F_ij_)= β_0_ + β_1_ ln(L_ij_) + β_2_ ln(D_i_) + β_3_ ln(R_ij_) |
|  | Wood | ln(W_ij_)= β_0_ + β_1_ ln(L_ij_) + β_2_ ln(D_i_) |

j^th^ branch on the i^th^ tree

L= branch length (m), F= foliage mass (g), W= wood mass (g)

D = tree diameter at breast height (cm); B = branch diameter (cm); C= width of crown at widest axis (m); S = height where branch meets main stem (m); R = relative branch depth; H= tree height (m); G = height to first branch (m)

Table S3: Parameter values and evaluation statistics for the regression models used to predict branch length, foliage and branch wood for individual branches. Numbers in parentheses are standard errors of the parameter values. Corr. Factor = correction factor; PICO = lodgepole pine (*Pinus contorta*); PIPO = ponderosa pine (*P. ponderosa*); PSME = Douglas fir (*Pseudotsuga menziesii)*

| Species | Component | n | β_0_ | β_1_ | β_2_ | β_3_ | β_4_ | Corr. Factor | Adj, R^2^ | RMSE * |
| --- | --- | --- | --- | --- | --- | --- | --- | --- | --- | --- |
| PICO | Branch Length (m) | 289 | 0.53 (0.085) | 0.41 (0.019) | -0.025 (0.004) | 0.12 (0.022) |  | NA | 0.63 | 0.3 |
|  | Foliage (g) | 102 | 3.73 (0.111) | 1.80 (0.129) | 0.30 (0.064) | 0.67 (0.244) |  | 1.17 | 0.74 | 151.3 |
|  | Wood (g) | 102 | 3.21 (0.174) | 1.78 (0.091) | 0.87 (0.121) | 0.40 (0.072) |  | 1.05 | 0.93 | 155.9 |
| PIPO | Branch Length (m) | 182 | 0.30 (0.129) | 0.26 (0.013) | 0.099 (0.024) | -0.082 (0.015) | 0.24 (0.083) | NA | 0.87 | 0.5 |
|  | Foliage (g) | 51 | 2.80 (0.575) | 1.99 (0.128) | 0.16 (0.210) | -0.75 (0.211) | -0.21 (0.181) | 1.12 | 0.91 | 1262.2 |
|  | Wood (g) | 51 | 3.41 (0.172) | 2.65 (0.077) | 0.086 (0.084) | 0.16 (0.148) |  | 1.04 | 0.98 | 4810.5 |
| PSME | Branch Length (m) | 275 | 0.51 (0.085) | 0.40 (0.015) | -0.011 (0.003) | 0.12 (0.037) |  | NA | 0.75 | 0.5 |
|  | Foliage (g) | 53 | 3.34 (0.415) | 1.73 (0.126) | 0.69 (0.142) | 0.40 (0.201) |  | 1.12 | 0.85 | 305.8 |
|  | Wood (g) | 53 | 3.53 (0.359) | 2.45 (0.114) | 0.53 (0.115) |  |  | 1.10 | 0.93 | 468.08 |

* RMSE values are in original units (either m for length or g for foliage and wood; not log transformed)

Table S4: List of predictor variable rasters considered for mapping biomass and references for each variable.

| Predictor Variable | References |
| --- | --- |
| **Topography and geomorphology** | Evans et al., [23] |
| Elevation and slope | Gesch et al., [20]; Gesch, [19] |
| Eastness and northness | Kumar et al., [21] |
| Compound topographic index | Gessler et al., [30] |
| Heat load index | McCune and Keon, [31] |
| Standard, profile, and planform curvature | Moore et al., [32]; Zevenbergen and Thorne, [33] |
| Roughness (3x3, 6x6, 9x9,12x12, 15x15, 21x21, 27x27) | Riley et al., [34]; Blaszczynski [35] |
| Slope position (3x3, 6x6, 9x9,12x12, 15x15, 21x21, 27x27) | De Reu [36]; Guisan et al., [37] |
| **Spectral** |  |
| ETM+ bands 1-7 |  |
| Soil-adjusted vegetation index (SAVI) | Huete, [38] |
| Normalized difference vegetation index (NDVI) | Rouse et al., [39] |
| Corrected NDVI | Nemani et al., [40] |
| Enhanced vegetation index (EVI) | Liu and Huete, [41] |
| Moisture stress index (MSI) | Rock et al., [42] |
| Second modified soil-adjusted vegetation index (MSAVI2) | Qi et al., [43] |
| Normalized difference infrared index (NDII) | Hardisky et al., [44] |
| Green normalized difference vegetation index (GNDVI) | Gitelson et al., [45] |
| Normalized difference water index (NDWI) | McFeeters, [46] |
| NDWI and NDII with ETM+ band 7 substituted for ETM+ band 5 | Ji et al., [47] |
| Tasseled Cap brightness, greenness, and wetness | Huang et al., [48] |
| Tasseled Cap distance | Duane et al., [49] |
| Tasseled Cap angle | Powell et al., [50] |
| **Texture** |  |
| Mean, variance, homogeneity, contrast, dissimilarity, entropy, second moment, and correlation for ETM+ bands 1, 2, 3, 4, 5, and 7 on a 3x3, 5x5, 7x7, 9x9, and 11x11 window and a (1,1) shift | Zvoleff, [12] |
| **Climate (1961-1990 normals)** |  |
| Chilling degree-days (degree-days below 0°C) | Wang et al., [24] |
| Growing degree-days (degree-days above 5°C) | Wang et al., [24] |
| Heating degree-days (degree-days below 18°C) | Wang et al., [24] |
| Cooling degree-days (degree-days above 18°C) | Wang et al., [24] |
| Frost-free period | Wang et al., [24] |
| Mean annual precipitation | Wang et al., [24] |
| Mean annual solar radiation | Wang et al., [24] |
| Mean annual temperature | Wang et al., [24] |
| Mean coldest month temperature | Wang et al., [24] |
| May to September precipitation | Wang et al., [24] |
| Number of frost-free days | Wang et al., [24] |
| Mean warmest month temperature | Wang et al., [24] |
| Precipitation as snow between August in previous year and July in current year | Wang et al., [24] |
| Temperature difference between mean warmest month temperature and mean coldest month temperature | Wang et al., [24] |
| Summer heat-moisture index | Wang et al., [24] |
| Annual heat-moisture index | Wang et al., [24] |

Table S5. Predictor variables used in the final random forest model for biomass maps made from each set of allometric equations: local (equations presented in this study), Jenkins et al. [8], and FIA-CRM. Variables are listed by order of importance, with the first variable being the most important. See Table 4, Additional File 1 for acronym definitions.

| Allometric Equations | Predictor Variables |
| --- | --- |
| Local | ETM+ band 1, NDII, elevation, ETM+ band 5 3x3 second moment, ETM+ band 6, precipitation as snow, northness, EVI, NDVI, mean annual solar radiation, slope position (6x6) |
| Jenkins et al. [8] | NDII, elevation, ETM+ band 1, Tasseled Cap angle, May to September precipitation, ETM+ band 1 5x5 entropy, mean annual solar radiation, ETM+ band 4 11x11 homogeneity, EVI, northness |
| FIA-CRM | NDII, ETM + band 2 5x5 mean, precipitation as snow, elevation, ETM+ band 5 3x3 second moment, EVI, ETM+ band 4 9x9 contrast, northness, slope position (6x6) |

Table S6: Comparison of DBH and height between destructively sampled trees presented in this study (Local trees), trees used from the Legacy Tree database (Legacy trees), and trees measured in the FIA plots. PSME = Douglas fir (*Pseudotsuga menziesii)*; PICO = lodgepole pine (*Pinus contorta*); PIPO = ponderosa pine (*P. ponderosa*)

|  | Species | Legacy trees | Local trees | FIA Plot Trees |
| --- | --- | --- | --- | --- |
| Number of trees sampled | PSME | 63 | 10 | 811 |
|  | PICO | 114 | 20 | 9601 |
|  | PIPO | 108 | 10 | 593 |
| Mean DBH (cm) | PSME | 14.1 | 24.9 | 21.5 |
|  | PICO | 11.4 | 16.3 | 19.9 |
|  | PIPO | 12.7 | 34.0 | 23.2 |
| Max DBH (cm) | PSME | 39.6 | 46.6 | 78.7 |
|  | PICO | 32.0 | 29.9 | 62.0 |
|  | PIPO | 36.6 | 61.8 | 64.3 |
| Min DBH (cm) | PSME | 1.5 | 2.4 | 2.5 |
|  | PICO | 1.0 | 2.5 | 2.5 |
|  | PIPO | 0.8 | 4.9 | 2.5 |
| Mean height (m)* | PSME | 8.0 | 12.7 | 11.7 |
|  | PICO | 7.0 | 12.2 | 13.9 |
|  | PIPO | 6.8 | 11.3 | 10.3 |
| Max height (m) | PSME | 19.5 | 19.7 | 34.1 |
|  | PICO | 16.2 | 21.1 | 31.1 |
|  | PIPO | 19.2 | 16.8 | 29.9 |
| Min height (m) | PSME | 1.8 | 3.2 | 1.8 |
|  | PICO | 1.5 | 3.2 | 2.1 |
|  | PIPO | 1.4 | 3.0 | 2.1 |

* Height was not reported for 19 lodgepole pine trees in the Legacy Tree database.

*Additional File 1 References*

1. Poudel KP, Temesgen H. Methods for estimating aboveground biomass and its components for Douglas-fir and lodgepole pine trees. Can J For Res. 2016;46:77–87.

2. Chung W, Evangelista P, Anderson N, Vorster A, Han H, Poudel K, et al. Estimating Aboveground Tree Biomass for Beetle-Killed Lodgepole Pine in the Rocky Mountains of Northern Colorado. For Sci. 2017;63:413–9.

3. Temesgen H, Monleon V, Weiskittel A, Wilson D. Sampling strategies for efficient estimation of tree foliage biomass. For Sci. 2011;57:153–63.

4. Kershaw JAJ, Maguire DA. Crown structure in western hemlock, Douglas-fir, and grand fir in western Washington: trends in branch-level mass and leaf area. Can J For Res. 1995;25:1897–912.

5. Poudel KP, Temesgen H, Gray AN. Evaluation of sampling strategies to estimate crown biomass. For Ecosyst. 2015;2.

6. Dormann CF, Elith J, Bacher S, Buchmann C, Carl G, Carré G, et al. Collinearity: A review of methods to deal with it and a simulation study evaluating their performance. Ecography (Cop). 2013;36:27–46.

7. Baskerville GL. Use of Logarithmic Regression in the Estimation of Plant Biomass. Can J For Res. 1972;2:49–53.

8. Jenkins JC, Chojnacky DC, Heath LS, Birdsey RA. National-scale biomass estimators for United States tree species. For Sci. 2003;49:12–35.

9. Phalla T, Ota T, Mizoue N, Kajisa T, Yoshida S, Vuthy M, et al. The Importance of Tree Height in Estimating Individual Tree Biomass while Considering Errors in Measurements and Allometric Models. AGRIVITA J Agric Sci. 2018;40:131–40.

10. Raile GK. Estimating stump volume. RP-NC-224. St. Paul, MN: U.S. Department of Agriculture, Forest Service, North Central Forest Experiment Station; 1982.

11. Heath LS, Hansen MH, Smith JE, Smith WB, Miles PD. Investigation into Calculating Tree Biomass and Carbon in the FIADB Using a Biomass Expansion Factor Approach. In: McWilliams W, Moisen G, Czaplewski R, editors. 2008 For Invent Anal Symp USDA For Serv Proceddings - RMRS-P-56. Park City, UT: U.S. Department of Agriculture, Forest Service, Rocky Mountain Research Station; 2009.

12. Zvoleff A. glcm: Calculate Textures from Grey-Level Co-Occurrence Matrices (GLCMs). 2016.

13. Kelsey KC, Neff JC. Estimates of aboveground biomass from texture analysis of landsat imagery. Remote Sens. 2014;6:6407–22.

14. Zhao P, Lu D, Wang G, Wu C, Huang Y, Yu S. Examining Spectral Reflectance Saturation in Landsat Imagery and Corresponding Solutions to Improve Forest Aboveground Biomass Estimation. Remote Sens. 2016;8.

15. Lu D, Batistella M. Exploring TM Image Texture and its Relationships with Biomass Estimation in Rondônia, Brazilian Amazon. Acta Amaz. 2005;35:249–57.

16. Lu D. Aboveground biomass estimation using Landsat TM data in the Brazilian Amazon. Int J Remote Sens. 2005;26:2509–25.

17. Swetnam TL, Brooks PD, Barnard HR, Harpold AA, Gallo EL. Topographically driven differences in energy and water constrain climatic control on forest carbon sequestration. Ecosphere. 2017;8.

18. Evans JS, Cushman SA. Gradient modeling of conifer species using random forests. Landsc Ecol. 2009;24:673–83.

19. Gesch DB. The National Elevation Dataset. In: Maune D, editor. Digit Elev Model Technol Appl DEM User’s Man. Second. Bethesda, Maryland: American Society for Photogrammetry and Remote Sensing; 2007. p. 99–118.

20. Gesch D, Oimoen M, Greenlee S, Nelson C, Steuck M, Tyler D. The National Elevation Dataset. Photogramm Eng Remote Sens. 2002;68.

21. Kumar S, Stohlgren TJ, Chong GW. Spatial heterogeneity influences native and nonnative plant species richness. Ecology. 2006;87:3186–99.

22. Environmental Systems Research Institute. ArcGIS. Redlands, CA; 2017.

23. Evans JS, Oakleaf J, Cushman SA, Theobald D. An ArcGIS Toolbox for Surface Gradient and Geomorphometric Modeling. 2014.

24. Wang T, Hamann A, Spittlehouse D, Carroll C. Locally downscaled and spatially customizable climate data for historical and future periods for North America. PLoS One. 2016;11.

25. Genuer R, Poggi J, Tuleau-Malot C. VSURF : An R Package for Variable Selection Using Random Forests. R J. 2015;7:19–33.

26. Friedman JH. Greedy Function Approximation : A Gradient Boosting Machine. Ann Stat. 2001;29:1189–232.

27. Sánchez Meador A. Legacy Tree Data [Internet]. 2007 [cited 2019 Feb 19]. Available from: Legacytreedata.org

28. Reid CPP. Effects of clearcutting on nutrient cycling in lodgepole pine forests. [Fort Collins, CO]: Colorado State University; 1974.

29. Tossey GB. Biomass Equations of Small Trees of Four Rocky Mountain Species with Reference to Habitat. [Fort Collins, CO]: Colorado State University; 1982.

30. Gessler PE, Moore ID, Mckenzie NJ, Ryan PJ. Soil-landscape modelling and spatial prediction of soil attributes. Int J Geogr Inf Syst. 1995;9:421–32.

31. McCune B, Keon D. Equations for potential annual direct incident radiation and heat load. J Veg Sci. 2002;13:603–6.

32. Moore ID, Grayson RB, Ladson AR. Digital terrain modelling: a review of hydrological, geomorphological, and biological applications. Hydrol Process. 1991;5:3–30.

33. Zevenbergen LW, Thorne CR. Quantitative analysis of land surface topography. Earth Surf Process Landforms. 1987;12:47–56.

34. Riley SJ, DeGloria SD, Elliot R. A Terrain Ruggedness Index That Quantifies Topographic Heterogeneity. Intermt J Sci. 1999;5:23–7.

35. Blaszczynski JS. Landform Characterization with Geographic Information Systems. Photogramm Eng Remote Sens. 1997;63:183–91.

36. Reu J De, Bourgeois J, Bats M, Zwertvaegher A, Gelorini V, Smedt P De, et al. Application of the topographic position index to heterogeneous landscapes. Geomorphology. 2013;186:39–49.

37. Guisan A, Weiss SB, Weiss AD. GLM versus CCA spatial modeling of plant species distribution. Plant Ecol. 1999;143:107–22.

38. Huete AR. A Soil-Adjusted Vegetation Index (SAVI). Remote Sens Environ. 1988;309:295–309.

39. Rouse JW, Haas RH, Schell JA, Deering DW. Monitoring vegetation systems in the Great Plains with ERTS. NASA Spec Publ. 1974;351:309–17.

40. Nemani R, Pierce L, Running S, Band L. Forest ecosystem processes at the watershed scale: sensitivity to remotely-sensed Leaf Area Index estimates. Int J Remote Sens. 1993;14:2519–34.

41. Liu HQ, Huete A. A Feedback Based Modificatoin of the NDVI to Minimize Canopy Background and Atmospheric Noise. IEEE Trans Geosci Remote Sens. 1995;33:457–65.

42. Rock BN, Williams DL, Vogelmann JE. Field and airborne spectral characterization of suspected damage in red spruce (Picea rubens) from Vermont. Mach Process Remote Sensed Data Symp. West Lafayette, Indiana; 1985. p. 71–81.

43. Qi J, Chehbouni A, Huete AR, Kerr YH, Sorooshian S. A Modified Soil Adjusted Vegetation Index. Remote Sens Environ. 1994;48:119–26.

44. Hardisky MA, Klemas V, Smart RM. The Influence of Soil Salinity, Growth Form, and Leaf Moisture on-the Spectral Radiance of Spartina alterniflora Canopies. Photogramm Eng Remote Sens. 1983;49:77–83.

45. Gitelson AA, Kaufman YJ, Merzlyak MN. Use of a Green Channel in Remote Sensing of Global Vegetation from EOS-MODIS. Remote Sens Environ. 1996;58:289–98.

46. McFeeters SK. The use of the Normalized Difference Water Index (NDWI) in the delineation of open water features. Int J Remote Sens. 1996;17:1425–32.

47. Ji L, Wylie BK, Nossov DR, Peterson B, Waldrop MP, McFarland JW, et al. Estimating aboveground biomass in interior Alaska with Landsat data and field measurements. Int J Appl Earth Obs Geoinf. 2012;18:451–61.

48. Huang C, Wylie B, Yang L, Homer C, Zylstra G. Derivation of a tasselled cap transformation based on Landsat 7 at-satellite reflectance. Int J Remote Sens. 2002;23:1741–8.

49. Duane M V, Cohen WB, Campbell JL, Hudiburg T, Turner DP, Weyermann DL. Implications of Alternative Field-Sampling Designs on Landsat-Based Mapping of Stand Age and Carbon Stocks in Oregon Forests. For Sci. 2010;56:405–16.

50. Powell SL, Cohen WB, Healey SP, Kennedy RE, Moisen GG, Pierce KB, et al. Quantification of live aboveground forest biomass dynamics with Landsat time-series and field inventory data: A comparison of empirical modeling approaches. Remote Sens Environ. 2010;114:1053–68.
