# Supplementary material for: Variability and uncertainty in forest biomass estimates from the tree to landscape scale: the role of allometric equations
Source: Carbon Balance Manag. 2020 May 14;15:8. doi: 10.1186/s13021-020-00143-6 (PMC7227279; doi:10.1186/s13021-020-00143-6)
Supplement: Supplementary file 2 — Additional file 2. Results of destructive sampling and oven drying; Tables and figures presenting mass and moisture content of each component from destructive sampling of Douglas-fir, lodgepole pine, and ponderosa pine. [file 13021_2020_143_MOESM2_ESM.docx]

*Additional File 2: Results of destructive sampling and oven drying*

Tables and figures presenting mass and moisture content of each component from destructive sampling of Douglas-fir, lodgepole pine, and ponderosa pine.

Table S1: Moisture content for the species and components in this study. Moisture content is presented as a percentage of the wet weight. The “n” for each component is the number of samples oven dried to obtain the moisture content.

| Species | Component | n | Mean (%) | SD (%) | Min (%) | Max (%) |
| --- | --- | --- | --- | --- | --- | --- |
| Douglas-Fir | Bole | 75 | 44.3 | 5.2 | 34.2 | 54.4 |
|  | Bark | 75 | 36.3 | 8.9 | 18.4 | 56.4 |
|  | Branch | 58 | 42.2 | 7.7 | 21.2 | 83.8 |
|  | Dead Branch | 14 | 8.7 | 7.3 | 2.1 | 33.1 |
|  | Foliage | 59 | 53.0 | 4.3 | 27.4 | 60.3 |
| Lodgepole Pine | Bole | 132 | 52.4 | 7.5 | 27.9 | 66.1 |
|  | Bark | 131 | 52.0 | 8.7 | 28.5 | 66.6 |
|  | Branch | 97 | 44.2 | 6.6 | 12.6 | 67.9 |
|  | Dead Branch | 10 | 7.9 | 0.9 | 6.9 | 9.1 |
|  | Foliage | 97 | 48.3 | 6.9 | 11.2 | 69.7 |
| Ponderosa Pine | Bole | 72 | 57.6 | 3.2 | 47.5 | 63.4 |
|  | Bark | 72 | 33.7 | 7.3 | 13.1 | 57.8 |
|  | Branch | 60 | 41.6 | 9.5 | 4.9 | 64.3 |
|  | Dead Branch | 13 | 6.7 | 2.6 | 3.9 | 12.8 |
|  | Foliage | 58 | 49.6 | 5.4 | 25.0 | 69.0 |

* A species-specific, whole-tree water percentage was calculated as the mean of each component’s moisture content weighted by the percentage of tree biomass in each component (Figure 1, Additional File 2). These values were used to convert trees from the Legacy Database that only reported wet mass to dry mass. The whole-tree water percentages were 44.20% for Douglas-fir, 48.45% for lodgepole pine, and 47.53% for ponderosa pine.

** The subsampled branches from the three smallest lodgepole pine trees were too small to obtain reliable water weights, so were not used in the moisture content calculations. This was also the case for one subsampled Douglas-fir branch and one ponderosa pine branch.


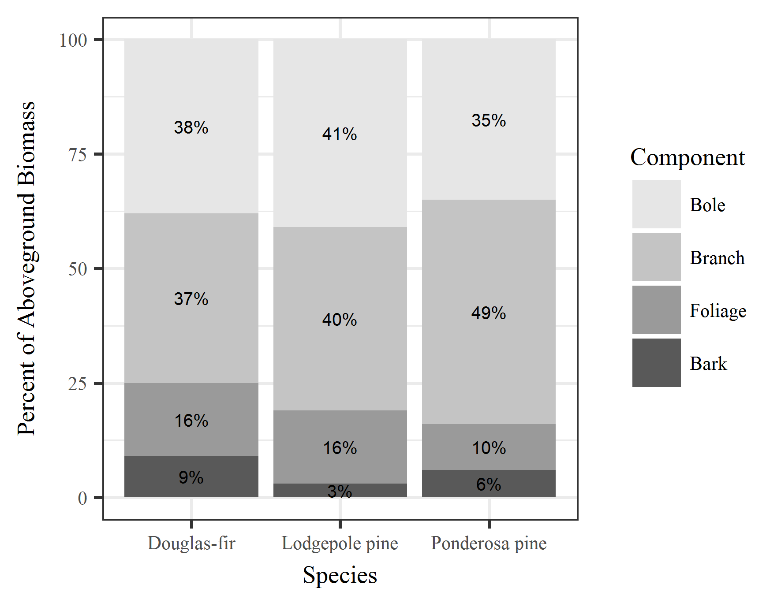


Fig. S1. The mean percentage of aboveground biomass held in each component for the destructively sampled trees.
