# Supplementary material for: Variability and uncertainty in forest biomass estimates from the tree to landscape scale: the role of allometric equations
Source: Carbon Balance Manag. 2020 May 14;15:8. doi: 10.1186/s13021-020-00143-6 (PMC7227279; doi:10.1186/s13021-020-00143-6)
Supplement: Supplementary file 3 — Additional file 3. Biomass variability between allometric equations; Tables and figures characterizing tree and plot-level biomass differences between the three allometric equations evaluated in this study. [file 13021_2020_143_MOESM3_ESM.docx]

*Additional File 3: Biomass variability between allometric equations*

Tables and figures characterizing tree and plot-level biomass differences between the three allometric equations evaluated in this study.

Table S1: Summary of individual tree biomass differences between different allometric equations. Differences are shown for all sizes (All) and by diameter at breast height bins. The denominators for calculating relative differences are the local biomass estimates for “Local-Jenkins” and “Local – FIA-CRM” and is Jenkins biomass for “Jenkins – FIA-CRM.”

|  | | | Local - Jenkins | | Local – FIA-CRM | | Jenkins – FIA-CRM | |
| --- | --- | --- | --- | --- | --- | --- | --- | --- |
| Species | Component | Diameter Range (cm) | Mean Diff (kg) | Mean Relative Diff (%) | Mean Diff (kg) | Mean Relative Diff (%) | Mean Diff (kg) | Mean Relative Diff (%) |
| Douglas-Fir (202) | Total | All | -88.9 | -34.3 | 18.8 | 29.3 | 107.7 | 47.8 |
|  |  | 2.5 - 20 | -17.2 | -23.5 | 24.7 | 46.3 | 41.9 | 56.1 |
|  |  | 20 – 40 | -97.4 | -43.4 | 19.2 | 12.8 | 116.6 | 39.0 |
|  |  | 40 - 60 | -487.6 | -61.6 | -8.3 | -1.0 | 479.3 | 37.2 |
|  |  | 60 - 80 | -1605.5 | -75.2 | -237.7 | -9.8 | 1367.8 | 37.3 |
|  | Bole and Bark | All | -91.7 | -47.2 | -5.5 | 17.8 | 95.2 | 47.6 |
|  |  | 2.5 - 20 | -16.4 | -28.6 | 18.8 | 41.2 | 38.5 | 57.7 |
|  |  | 20 – 40 | -101.2 | -63.2 | -7.1 | -0.2 | 94.1 | 38.8 |
|  |  | 40 - 60 | -508.5 | -92.2 | -114.7 | -21.4 | 393.9 | 36.8 |
|  |  | 60 - 80 | -1658.3 | -112.2 | -510.3 | -33.1 | 1148.0 | 37.3 |
|  | Branch | All | 3.1 | 0.4 | 25.7 | 57.0 | 22.6 | 54.8 |
|  |  | 2.5 - 20 | -0.8 | -6.5 | 10.1 | 64.4 | 10.9 | 65.3 |
|  |  | 20 – 40 | 3.8 | 6.0 | 26.3 | 50.4 | 22.5 | 45.6 |
|  |  | 40 - 60 | 20.9 | 10.2 | 106.4 | 51.3 | 85.5 | 44.4 |
|  |  | 60 - 80 | 52.9 | 8.9 | 272.6 | 47.0 | 219.8 | 41.5 |
|  | Foliage | All | 1.8 | 17.7 | NA | NA | NA | NA |
|  |  | 2.5 - 20 | 1.8 | 22.6 | NA | NA | NA | NA |
|  |  | 20 – 40 | 3.1 | 15.1 | NA | NA | NA | NA |
|  |  | 40 - 60 | -3.0 | -3.3 | NA | NA | NA | NA |
|  |  | 60 - 80 | -40.7 | -22.7 | NA | NA | NA | NA |
| Lodgepole Pine (108) | Total | All | 31.7 | 18.7 | 41.7 | 28.3 | 10.0 | 11.6 |
|  |  | 2.5 - 20 | 11.9 | 18.8 | 21.3 | 33.9 | 9.3 | 18.2 |
|  |  | 20 – 40 | 51.3 | 18.2 | 57.2 | 20.0 | 6.0 | 2.0 |
|  |  | 40 - 60 | 268.9 | 25.7 | 417.1 | 39.6 | 148.2 | 18.7 |
|  |  | 60 - 80 | 845.3 | 32.8 | 1439.7 | 55.9 | 594.4 | 34.4 |
|  | Bole and Bark | All | 13.0 | -1.2 | 21.7 | 12.7 | 7.6 | 10.9 |
|  |  | 2.5 - 20 | -2.5 | -9.8 | 5.2 | 13.2 | 7.8 | 18.5 |
|  |  | 20 – 40 | 26.4 | 9.9 | 30.0 | 11.0 | 3.6 | 1.3 |
|  |  | 40 - 60 | 250.8 | 27.8 | 368.5 | 40.6 | 117.7 | 17.9 |
|  |  | 60 - 80 | 878.3 | 37.8 | 1308.6 | 56.3 | 430.3 | 29.8 |
|  | Branch | All | 19.7 | 52.7 | 22.7 | 60.5 | 3.0 | 15.6 |
|  |  | 2.5 - 20 | 15.6 | 59.1 | 18.3 | 69.7 | 2.6 | 24.2 |
|  |  | 20 – 40 | 24.8 | 46.1 | 27.3 | 49.8 | 2.4 | 4.6 |
|  |  | 40 - 60 | 18.0 | 15.1 | 48.5 | 38.2 | 30.5 | 25.6 |
|  |  | 60 - 80 | -33.1 | -15.4 | 131.0 | 61.0 | 164.1 | 66.2 |
|  | Foliage | All | 4.2 | 39.8 | NA | NA | NA | NA |
|  |  | 2.5 - 20 | 3.9 | 49.4 | NA | NA | NA | NA |
|  |  | 20 – 40 | 4.8 | 27.9 | NA | NA | NA | NA |
|  |  | 40 - 60 | -3.8 | -8.1 | NA | NA | NA | NA |
|  |  | 60 - 80 | -30.4 | -42.4 | NA | NA | NA | NA |
| Ponderosa Pine (122) | Total | All | 26.3 | 8.9 | 101.7 | 48.8 | 75.5 | 43.6 |
|  |  | 2.5 - 20 | 4.2 | 8.0 | 31.8 | 56.4 | 27.6 | 52.5 |
|  |  | 20 – 40 | 26.3 | 8.8 | 118.9 | 43.6 | 92.5 | 38.1 |
|  |  | 40 - 60 | 152.0 | 14.3 | 405.7 | 39.8 | 253.6 | 29.7 |
|  |  | 60 - 80 | 477.3 | 20.2 | 405.2 | 17.1 | -72.1 | -3.8 |
|  | Bole and Bark | All | -46.2 | -21.0 | 15.2 | 26.8 | 65.7 | 42.3 |
|  |  | 2.5 - 20 | -4.2 | -4.3 | 20.1 | 46.1 | 25.5 | 51.5 |
|  |  | 20 – 40 | -51.8 | -30.9 | 23.0 | 18.5 | 74.8 | 37.8 |
|  |  | 40 - 60 | -253.5 | -52.6 | -43.3 | -7.5 | 210.2 | 29.6 |
|  |  | 60 - 80 | -631.5 | -66.6 | -882.7 | -93.1 | -251.2 | -15.9 |
|  | Branch | All | 79.0 | 56.5 | 95.0 | 79.3 | 16.0 | 49.2 |
|  |  | 2.5 - 20 | 10.4 | 43.0 | 17.4 | 77.0 | 7.1 | 58.3 |
|  |  | 20 – 40 | 78.1 | 63.3 | 95.9 | 80.1 | 17.8 | 44.8 |
|  |  | 40 - 60 | 405.6 | 75.4 | 449.0 | 84.0 | 43.4 | 34.3 |
|  |  | 60 - 80 | 1108.8 | 81.9 | 1287.9 | 95.1 | 179.1 | 72.9 |
|  | Foliage | All | 2.9 | -2.7 | NA | NA | NA | NA |
|  |  | 2.5 - 20 | -0.2 | -26.3 | NA | NA | NA | NA |
|  |  | 20 – 40 | 3.1 | 13.4 | NA | NA | NA | NA |
|  |  | 40 - 60 | 20.1 | 26.8 | NA | NA | NA | NA |
|  |  | 60 - 80 | 54.7 | 33.0 | NA | NA | NA | NA |


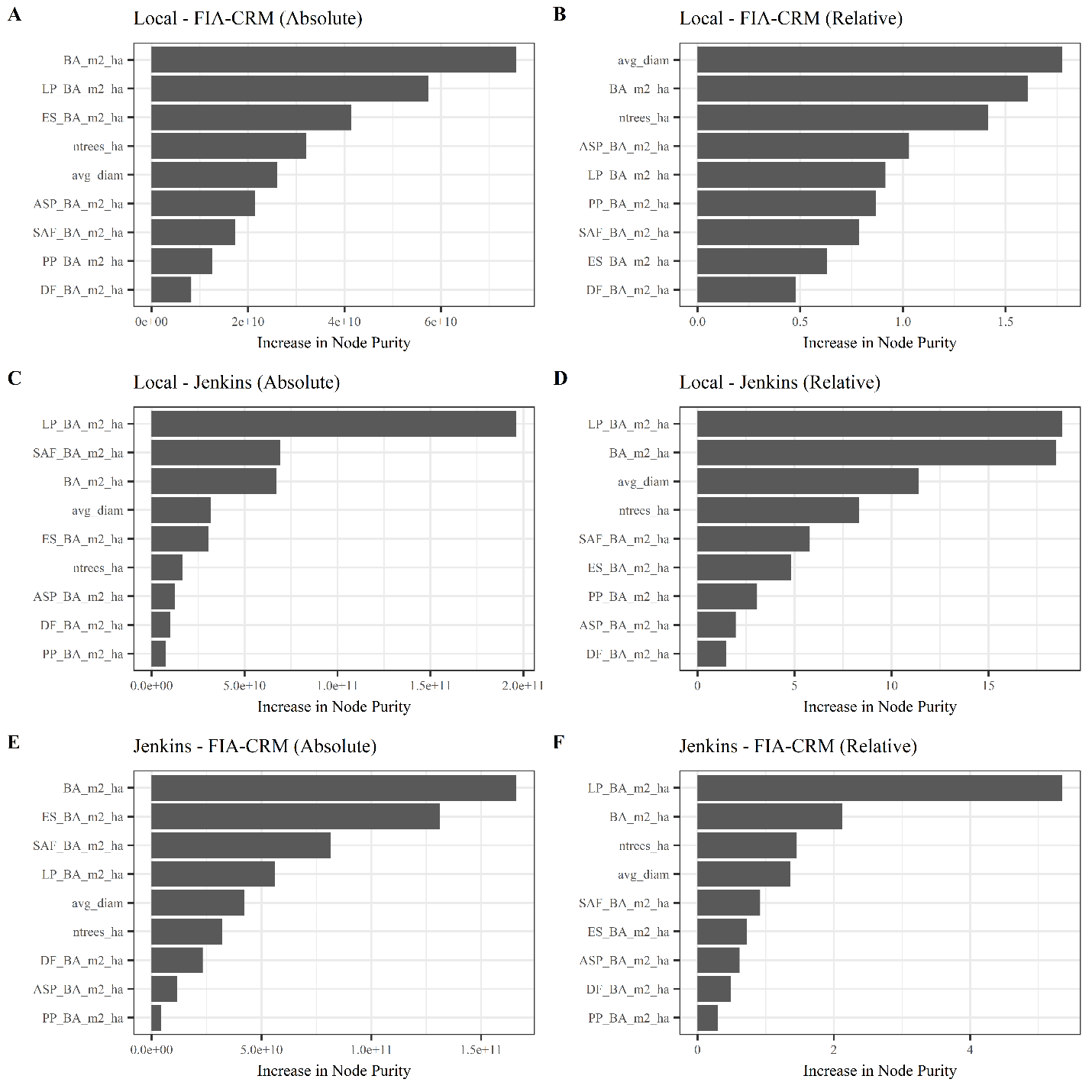


Fig. S1: Variable importance plots for random forests used to model differences in plot biomass estimates between different allometric equations estimates as a function of stand attributes. Both the absolute difference between plot biomass estimates was modeled (left column) as well as the relative difference (right column).

Allometric biomass equations compared: local = allometric equations presented in this study; Jenkins = allometric biomass equations from Jenkins et al. (2003); FIA-CRM = Forest Inventory and Analysis Component Ratio Method allometric biomass equations

Predictor variables considered: BA_m2_ha = total basal area (m^2^ ha^-1^); ntrees_ha = number of trees ha^-1^; avg_diam = average tree diameter (cm); LP_BA_m2_ha = lodgepole pine basal area (m^2^ ha^-1^); PP_BA_m2_ha = ponderosa pine basal area (m^2^ ha^-1^); DF_BA_m2_ha = Douglas-fir basal area (m^2^ ha^-1^); ES_BA_m2_ha = Engelmann spruce basal area (m^2^ ha^-1^); SAF_BA_m2_ha = subalpine fire basal area (m^2^ ha^-1^); ASP_BA_m2_ha = aspen basal area (m^2^ ha^-1^)


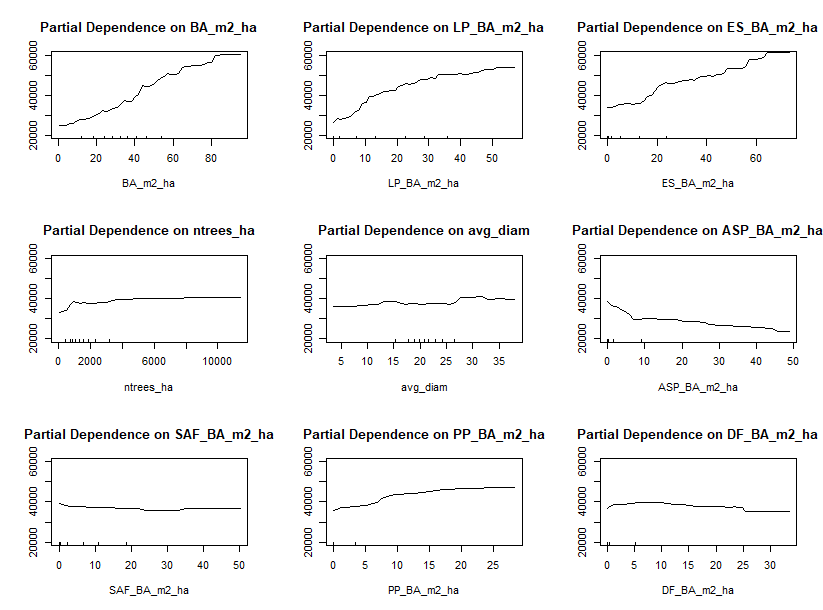


Fig. S2: Partial dependence plot from the random forests model of FIA plot biomass difference between local and FIA-CRM allometric equations as a function of stand attributes. Abbreviations of predictor variables considered are described in caption for Figure 1, Additional File 3.


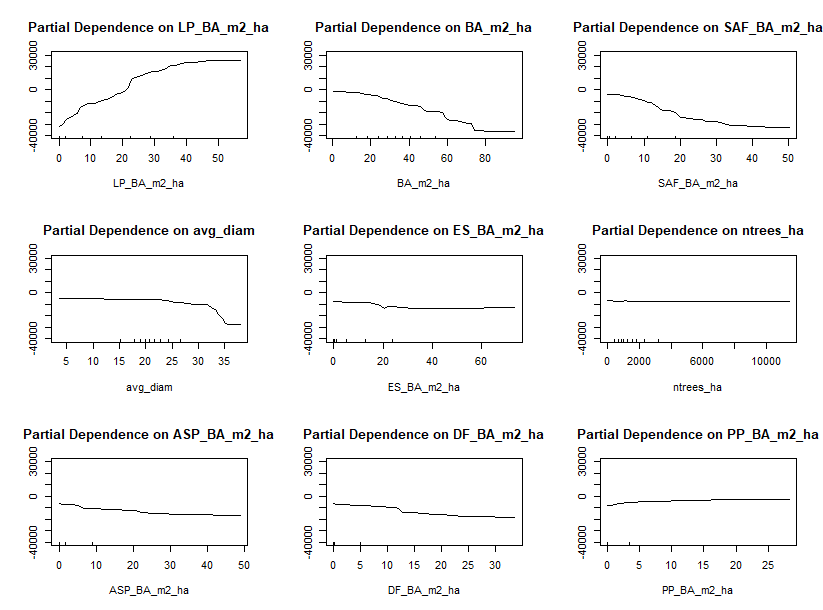


Fig. S3: Partial dependence plot from the random forests model of FIA plot biomass difference between local and Jenkins et al. (2003) allometric equations as a function of stand attributes. Abbreviations of predictor variables considered are described in caption for Figure 1, Additional File 3.


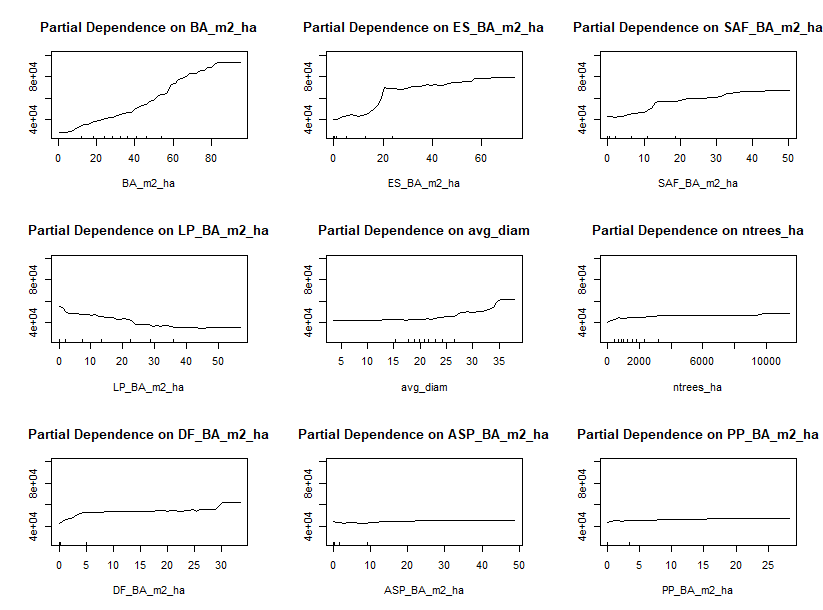


Fig. S4: Partial dependence plot from the random forests model of FIA plot biomass difference between Jenkins et al. (2003) and FIA-CRM allometric equations as a function of stand attributes. Abbreviations of predictor variables considered are described in caption for Figure 1, Additional File 3.

Table S2: Out-of-bag RMSE, percent RMSE of the mean, and pseudo R^2^ for random forests models run with plot biomass difference as the response variable and stand characteristics as predictor variables. Both relative and absolute model differences were modeled for the following allometric equations: local (presented in this study), Forest Inventory and Analysis Component Ratio Method (FIA-CRM), and Jenkins et al. (2003).

| Allometric Equations Compared | Relative or Absolute | RMSE | % RMSE (of mean) | Pseudo R^2^ |
| --- | --- | --- | --- | --- |
| Local – Jenkins | Absolute | 12.2 Mg ha ^-1^ | -178.6 | .8613 |
| Local – Jenkins | Relative | 34.8 % | -267.6 | .3903 |
| Local – FIA-CRM | Absolute | 17.7 Mg ha ^-1^ | 47.5 | .5668 |
| Local – FIA-CRM | Relative | 11.7 % | 40.6 | .4336 |
| Jenkins - FIA-CRM | Absolute | 21.4 Mg ha ^-1^ | 48.5 | .6633 |
| Jenkins - FIA-CRM | Relative | 12.2 % | 37.1 | .5529 |
